# The combined effects of sampling parameters on the sorbent tube sampling of phthalates in air

**DOI:** 10.1038/srep45677

**Published:** 2017-03-31

**Authors:** Sang-Hee Jo, Ki-Hyun Kim, Kyenghee Kwon

**Affiliations:** 1 Department of Civil and Environmental Engineering, Hanyang University, 222 Wangsimni-ro, Seoul, 04763, Korea; 2 College of Pharmacy, Dongkuk University, Goyang-si 10326, Korea

## Abstract

The adsorption properties of various sorbent materials were investigated to assess the factors affecting biases in the sorbent tube (ST) sampling of airborne phthalates. The recovery of phthalates was assessed critically in relation to four key sampling parameters: (1) three types of sorbent materials (quartz wool (QW), glass wool (GW), and quartz wool plus Tenax TA (QWTN)), (2) the concentration level of phthalate standards, (3) purge flow rate, and (4) purge volume for analysis based on a ‘sorbent tube-thermal desorption-gas chromatography-mass spectrometry (ST-TD-GC-MS)’ system. Among these parameters, the type of ST was the most influential in determining the recovery of phthalates. For a given ST type, the recovery of phthalates tends to improve with increases in the concentration level of standards. In case of QW and QWTN tubes, the breakthrough of phthalates was not observed up to the maximum purge volume (100 L) tested in this work; however, in case of GW, the recovery decreased drastically to 60% even at a purge volume of 1 L for low molecular weight phthalates. The results of our study demonstrate that accurate analysis of airborne phthalates can be achieved through proper control of key sampling parameters, particularly the choice of sorbent material.

Semi-volatile organic compounds (SVOCs) are classified as organic compounds with boiling points ranging from 240–260 to 380–400 °C^1^. According to this definition, the SVOC category is wide enough to include a list of organic pollutants such as pesticides, phthalates, fire retardants (polychlorinated biphenyls (PCBs) and polybrominated biphenyls (PBBs)), and polycyclic aromatic hydrocarbons (PAHs) commonly present in indoor and outdoor environments^2^. In recent years, concerns related to the exposure levels of SVOCs and associated human health risks (disruption of the human endocrine system) have risen due to the recognition of their ubiquitous presence in the indoor environment^3^,^4^. It was reported that phthalates were the most widely used plasticizers worldwide because of their large demand in industrial and consumer products; their annual use in Europe was estimated at 1 million tons^3^.

In light of the environmental significance of phthalate pollution, their emission concentrations and cycling have been intensively investigated in the recent past^5^. In this respect, the need for accurate quantitation of low (sub ppb) level phthalates in ambient air is in great demand. For the collection of airborne phthalates, the use of sampling filters (glass fiber filter (GFF), quartz fiber filter (QFF), polyurethane foam (PUF), and XAD^®^-2) has generally been recommended. After sampling, phthalates adsorbed onto the sampling filters are extracted via adequate solvents and concentrated using the Soxhlet extraction method^4^,^6^,^7^,^8^.

In recent years, use of the simple and efficient sorbent tube/thermal desorption (ST/TD) method has been proposed and exercised as an alternative to the aforementioned conventional methods based on complicated and time-consuming pretreatment procedures^9^,^10^. The ST/TD method is found to be efficient at reducing the bias involved in sampling phthalates by eliminating complicated pretreatment procedures like solvent extraction and pre-concentration and is also effective enough to lower their detection limits^2^. However, as the experimental validity of the ST/TD method has not yet sufficiently been verified, relatively little is known about the adsorption capacity (or breakthrough volume) of phthalates via sorbent materials used for TD analysis relative to the common sampling media (filters used for conventional sampling). Furthermore, the breakthrough is a key variable to assess the reliability of the ST method which can be affected by many variables such as the type of sorbent material, sampling volume, sampling flow rate, concentration level of the target analyte, and temperature^11^,^12^. Although breakthrough behavior is a critical parameter in the ST/TD-based quantitation of pollutants, such properties have not yet been sufficiently evaluated in relation to the recovery of phthalates against various sorbents used for these applications.

In our recent study, relative recovery in the ST/TD-based analysis of phthalates was investigated in reference to a direct injection method using a gas chromatography (GC)/mass spectrometry (MS) system^13^. In an effort to improve the quantitation procedure for phthalates, we extended our investigation to the quantitation techniques of airborne phthalates by focusing on the effect of various sampling parameters on ST/TD-GC/MS applications. To this end, the recovery of phthalates was assessed relative to three types of sorbent combinations ((1) quartz wool: QW, (2) glass wool: GW, and (3) quartz wool plus Tenax TA: QWTN) in association with other important sampling conditions (concentration level of phthalate standards, purge flow rate, and purge volume). Based on these experimental results, the potential effects of each sampling parameter in the TD-based quantitation of phthalates were examined in terms of recovery.

## Results and Discussion

### General adsorption properties of phthalates in terms of ST type

In the current study, the adsorption behavior of airborne phthalates was examined in relation to four sampling parameters based on an ST/TD-GC/MS system. Prior to a comparison of recovery with respect to sampling variables, the basic information of quality assurance (QA), including the calibration analysis results obtained via the reference purge method, is summarized in Table S1. The response factor (RF) values were measured by dividing the peak area data obtained from five-point calibration against the corresponding mass amount of target analyte. All calibration curves of the seven phthalates, derived for each type of ST (QW, GW, and QWTN), exhibited a fairly good linearity (R^2^ > 0.99) in the absolute mass range of 1 to 50 ng. The RF values of the GW tube for all target compounds were the smallest among all three types of STs. This poor adsorption capacity of the GW tube for the low molecular weight compounds (such as DMP and DEP) was consistent with what we observed in our recent study^13^. In terms of method detection limit (MDL), the results of six target phthalates (DMP, DEP, DBP, BBP, DEHA, and DOP) were determined at fairly low levels of 17.8 to 77.9 pg. These results were similar to or even lower than those values (11.7–6,950 pg) estimated via conventional sampling filters (quartz fiber filter, glass fiber filter, and XAD-2) as reported in earlier studies^4^,^7^,^14^. If our mass-based MDL values (pg) are converted into concentration terms (ng m^−3^) by assuming a sampling volume of 100 L, they correspond to 0.18 to 0.78 ng m^−3^. It should, however, be noted that due to the unavoidable contamination of DEHP during sample collection or preparation, its MDL was slightly higher (4.00 to 16.2 ng m^−3^) than those of the other six phthalates in all STs^15^.

The recovery results for seven phthalates obtained using QW, GW, and QWTN tubes are summarized in respective Tables 1, 2, and 3 in relation to the four sampling parameters investigated in this study (ST type, the concentration level of liquid standards, purge flow rate, and purge volume). To examine the effect of N_2_ purging, each L-WS was also analyzed just after injection into ST without N_2_ purge treatment (as a reference to N_2_ purging). First, the average recovery of all seven phthalates, when compared between three ST types (QW, GW, and QWTN tubes) without considering the effect of three other sampling parameters, was 92.1 ± 4.41% (Max: 106% and Min: 79.8%), 80.7 ± 28.7% (Max: 109% and Min: 1.05%), and 99.9 ± 4.75% (Max: 112% and Min: 88.3%), respectively. Based on this comparison, it seems that the QWTN tube recorded the maximum adsorption capacity from all target phthalates. A variation in recovery was maintained at the lowest level for the QWTN tube with respect to alterations in the purge conditions. The average recovery of the GW tube was the poorest among the three types of STs. In addition, their recovery values were distinguished based on purge volume and flow rate.

### Comparison of recovery with different purge volume

**Figure 1:**
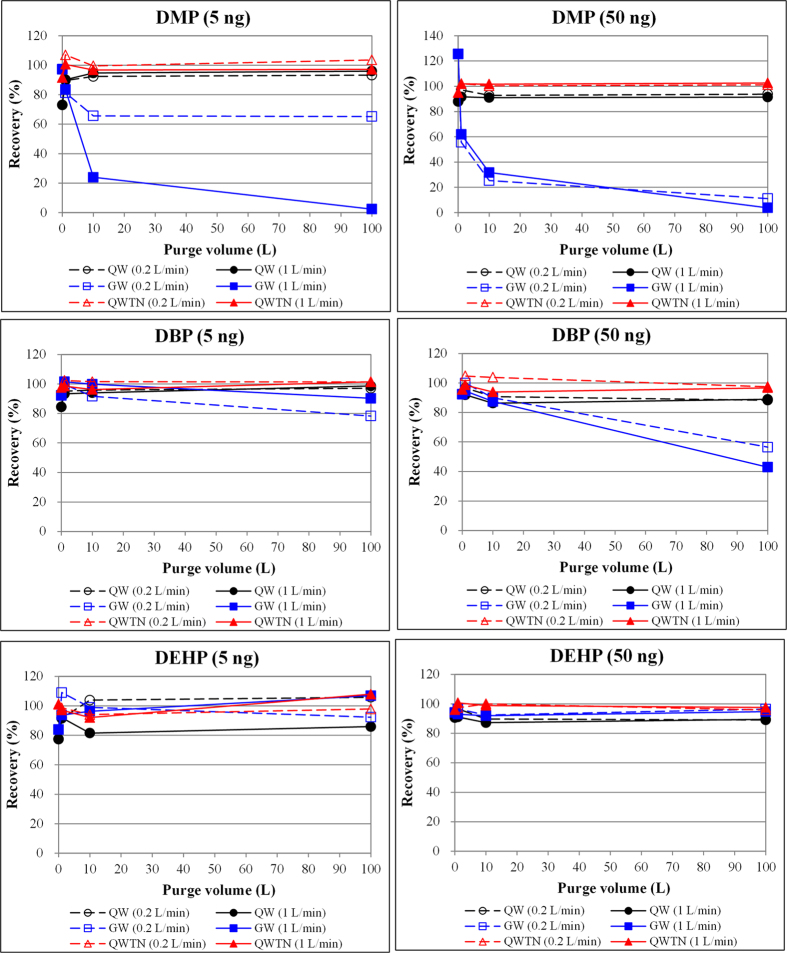
Comparison of recovery patterns (%) between various phthalates. Results are compared between sorbent tube types as a function of purge volume (up to 100 L).

The recovery of three representative phthalates with varying purge volumes is plotted in Fig. 1. In general, there was no signal of significant breakthrough for any of the target phthalates in QW and QWTN tubes with increased purge volume (of N_2_ gas) up to the maximum test volume of 100 L. In case of the QW tube, the recovery of phthalate compounds without purge treatment (purge time = 0 min) ranged from 73.0 to 96.7% (Table 1). The recoveries reached 84.9 to 98.9% after loading 1 L purge gas and remained constant until reaching a purge volume of 100 L. This enhanced recovery can be explained via the elimination of solvent residues in the ST, which then induces a reduction in the efficiency of adsorption-partitioning between analytes and sorbent materials^16^. The sampling efficiency of some phthalates (DEP, DBP, BBP, DEHP, and DOP) decreased to 85% after loading the QW tube with 100 L of gas at a flow rate of 1 L min^−1^. However, a nearly constant ST recovery was observed in the range of purging volume (1 to 100 L). Similarly, the recovery of all seven phthalates in the QWTN tube was retained at approximately 100% after the completion of purge treatment using 100 L N_2_ gas (Table 3).

This breakthrough behavior of SVOCs (phthalates) contrasts sharply with that of relatively light VOCs (with a molecular weight between 58.1 and 116 g mol^−1^) when tested with the Tenax TA adsorbent, which were directly affected by ST breakthrough at or above a 1 L purge volume (at 100 mL min^−1^ for 10 min)^16^. The breakthrough of phthalates was not observed in case of the two STs (QW and QWTN) up to the maximum purge volume (100 L) tested in this experiment. Note that there are clear distinctions in breakthrough volumes for the collection of common SVOCs (such as PAHs, PCBs, or organochlorine pesticides) between the ST/TD used in this work (a maximum testing volume of 0.1 m^3^) and conventional sampling methods (PUF and QFF filters: a common range of 300 to 10,000 m^3^)^17^.

Unlike other tube types, the adsorption characteristics of the GW tube were carefully distinguished in terms of the physicochemical properties of the target compounds (molecular weight or boiling point). In case of relatively low molecular weight compounds (DMP, DEP, and DBP), their recovery in the GW tube decreased exponentially with increasing purge volume (Figs 1 and S1). For DMP in particular, the lowest molecular weight compound in this study, recovery fell to 60% even at a purge volume of 1 L (Table 2). After the addition of 100 L of purge gas, the adsorption efficiency of DMP was reduced considerably (below 4%) at a purge flow rate of 1 L min^−1^. In contrast, no sample loss was observed in relatively high molecular weight compounds (BBP, DEHA, DEHP, and DOP) at a purge volume of up to 100 L in the GW tube. As such, adsorbent material made of glass wool (or fiber) is perceived as a reliable sample for the collection of particulate-phase SVOCs (organochlorine pesticides, PCBs, or PAHs)^5^. The adsorption efficiency of the GW tube was considerably low for the lower molecular weight compounds (such as DMP and DEP), which generally exists in the gas-phase in ambient air rather than in the particulate-phase^8^,^18^,^19^.

### Comparison of recovery as a function of phthalate concentration levels

**Figure 2:**
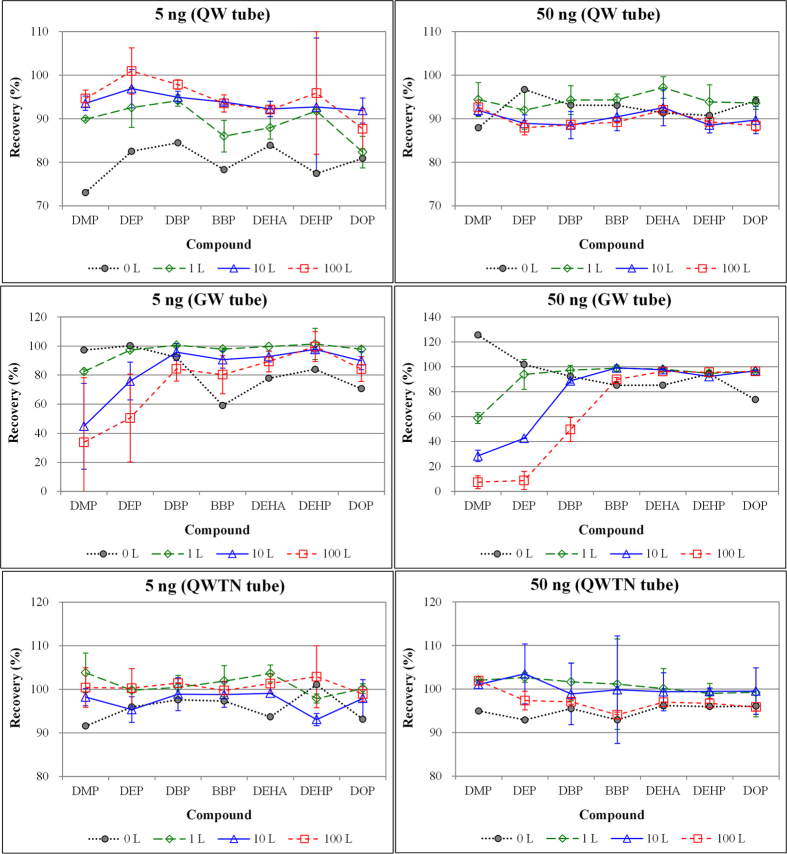
Comparison of recovery patterns (%) between various phthalates: Results are compared at two loading masses (5 vs. 50 ng) across the phthalate compounds.

In order to explore the adsorption properties of phthalates in relation to standard concentration levels, 1 μL standard samples at three concentrations (5, 20, and 50 ng μL^−1^) were injected independently into STs while supplying purge gas (1, 10, and 100 L) at flow rates of 0.2 and 1 L min^−1^. The recovery of seven target phthalates was compared in terms of absolute mass amount loaded onto each ST in Figs 2 and S2. A wide distribution of recovery between standard concentration levels was seen in the QW tube. The variation in recovery for phthalate compounds with low (5 ng μL^−1^) concentration levels was relatively large compared to high (50 ng μL^−1^) concentration levels. A wide variation in the recovery of the low molecular weight compounds (DMP, DEP, and DBP) was observed in the GW tube, regardless of standard concentration levels due to the ST breakthrough. However, the gap between the maximum and minimum recovery of the high molecular weight compounds (BBP, DEHA, DEHP, and DOP) decreased with the increases in standard concentration levels. The variation in phthalate recoveries in the QWTN tube was nearly constant, regardless of the concentration levels. This suggests that air sampling of phthalates can be reliably taken using a QWTN tube without being affected by differences in the concentration levels of phthalates relative to other ST types (QW or GW). As in our recovery data, the sampling efficiency of a sorbent tube made of polydimethylsiloxane (PDMS) and Tenax TA was not affected by the concentration levels of PAHs when spiked in the range of 0.25 to 8.0 ng^2^. Likewise, it was also reported that the breakthrough properties of Tenax TA sorbent for many light VOCs (aromatic hydrocarbons, aldehydes, and ketones) were free from detectable concentration levels (at ppb levels or mass loaded onto STs in the range of 37.3 to 559 ng)^12^.

### Comparison of recovery with different purge flow rate

In order to investigate the effect of purge flow rate on the recovery of each ST, the liquid standards of phthalates were purged at two contrasting purge rates of 0.2 and 1 L min^−1^ with the aid of N_2_ gas. The results of the recovery derived from these two purge flow rates are summarized in Table 4. The differences in recovery were assessed at a statistical significance of 95% (p < 0.05) for all compounds. The results are also plotted in relation to various purge flow rates in Fig. S3. First, the recovery of seven target phthalates (between two flow rates) is shown to be similar in the QW tube; however, differences in relative recovery were statistically significant (p < 0.05) between purge flow rates for DEHP, and not for the other six compounds (Table 4). In addition, there were no significant differences in recovery data across all GW tube results. The purge flow rate appears to be a negligible factor in terms of recovery for the collection of our target phthalates. In case of the QWTN tube, however, the recovery of phthalate compounds purged at the lower flow rate (0.2 L min^−1^) was larger than that of the higher flow rate (1 L min^−1^) across all concentration ranges (Fig. S3). Note that such differences are statistically significant for all compounds except DMP and DEHP. These results may reflect the adsorption properties of porous polymer adsorbents (Tenax TA), which generally exhibit optimum sampling performance at a flow rate of 50 to 200 mL min^−1^ 20. It was also found that the collection efficiencies of DEP and DBP via Tenax GR tube at the higher flow rate (200 mL min^−1^) were approximately 1.3 and 2.4 times greater than those measured at the lower flow rate (20 mL min^−1^) when the sampling duration of the target analyte was between 0 to 5 min^21^. As such, it has been experimentally demonstrated that the use of a specific flow rate (200 mL min^−1^) is desirable to optimize the sampling efficiency of the Tenax-like adsorbents. Nevertheless, the use of higher flow rates (above 200 mL min^−1^) is often inevitable for an extended sampling of semi-volatile (high boiling-point) compounds such as PCBs and PAHs in air^22^.

## Conclusions

In this study, the adsorption characteristics of airborne phthalates were investigated with respect to various sampling conditions based on ST-TD-GC-MS application. The performance of the ST recovery was assessed by controlling four sampling parameters: (1) ST type (QW, GW, and QWTN), (2) standard concentration levels (5, 20, and 50 ng μL^−1^), (3) purge flow rate (0.2 and 1 L min^−1^), and (4) purge volume (1, 10, and 100 L). The average recovery of seven target phthalates was 92.1 ± 4.41% (Max: 106% and Min: 79.8%), 80.7 ± 28.7% (Max: 109% and Min: 1.05%), and 99.9 ± 4.75% (Max: 112% and Min: 88.3%), when compared between three ST types without considering the effect of three other sampling parameters. For a given ST type, the recoveries of phthalates in the QW and GW tubes generally tend to improve with the increased concentration level of standards, while those in the QWTN tube were nearly constant, regardless of the concentration levels.

The breakthrough of phthalates was not observed up to the maximum test volume of 100 L in the QW and QWTN tubes; however, their recovery in the GW tube decreased exponentially with increasing purge volume for the relatively low molecular weight compounds (DMP, DEP, and DBP). In terms of purge flow rate, there were no significant differences in recovery obtained from the QW and GW tubes at a statistical criteria of 95% (p < 0.05). In contrast, differences between the purge flow rates in the QWTN tube were statistically significant for most phthalate compounds; recovery purged at the lower flow rate (0.2 L min^−1^) was larger than that at the higher rate (1 L min^−1^). Based on the recovery data in this work, the most influential factor in the recovery of airborne phthalates was the type of ST (or sorbent material). Consequently, QWTN is considered the best sorbent material in light of the adsorption efficiency of seven phthalates for extended sampling (average recovery after 100 L purge: QW: 91.9%, GW: 69.1%, and QWTN: 99.1%). In summary, we recommend using a QWTN tube at a low flow rate (0.2 L min^−1^) for the collection of airborne phthalates present in indoor and outdoor environments.

## Materials and Methods

### Preparation of liquid working standards for phthalates

For the ST/TD-GC/MS calibration analysis of phthalates, a standard mixture of seven phthalates (dimethyl phthalate (DMP), diethyl phthalate (DEP), dibutyl phthalate (DBP), benzyl butyl phthalate (BBP), di(2-ethylhexyl) adipate (DEHA), di(2-ethylhexyl) phthalate (DEHP), and di-n-octyl phthalate (DOP)) containing 1,000 μg mL^−1^ each was purchased and used as the primary standard (PS) (EPA 506 phthalate mix, Supelco, PA, USA). Their liquid phase working standards (L-WS) were prepared by diluting the PS with 100% methanol (J. T. Baker, PA, USA) at five concentration levels (1, 5, 10, 20, and 50 ng μL^−1^) in 2 mL vials. Basic information regarding the seven target phthalates and their relevant properties is presented in Table S2. To prevent sample contamination during the preparation of the L-WS, all laboratory equipment (liquid syringe, vials, or pipette tips) was cleaned using methanol and stored in an oven maintained at 200 °C prior to use.

### Preparation of sorbent tubes for the collection of phthalates in air

To compare the ST adsorption capacities of the seven phthalates, three types of sorbent packing (QW, GW, and QWTN) were prepared and tested for comparative purposes. Note that for this test, QW and GW were selected for their considerably good sorption capacity for SVOCs like PAHs^23^. QWTN tubes were also included, as they have been used widely in many previous studies for the collection of SVOCs including phthalates^2^,^9^,^10^,^24^. To prepare the three types of STs, an empty quartz tube (6.35 mm × 90 mm) was packed individually, containing: (1) 10 mg of QW (Grace, IL, USA), (2) 10 mg of GW (Supelco, PA, USA), and (3) 50 mg of Tenax TA (35–60 mesh, Markes International, UK) and 10 mg of QW (5 mg each at the initial and end of the Tenax TA). After the preparation, all STs were cleaned by supplying pure N_2_ gas at 100 mL min^−1^ at 320 °C for 24 h.

### Experimental approach and instrumental setup for the TD-GC-MS

For a comparative analysis of the adsorption properties between the seven phthalates, the effect of the ST types (QW, GW, and QWTN) was first assessed as the primary sampling variable in terms of relative recovery for each target compound. The reliability of this method has been examined further by controlling three other sampling parameters: the concentration levels of L-WS (5, 20, and 50 ng μL^−1^), purge flow rates (0.2 and 1 L min^−1^), and purge volumes (1, 10, and 100 L) of nitrogen gas. To understand the adsorption capacities of the ST method against the target compounds present in air, their gaseous standards are loaded at varying quantities to assess the breakthrough capacities. Considering the difficulties in preparing the gaseous standards of SVOC-like phthalates (at room temperature), a gas purging technique was applied to generate their gas phase standards (from liquid phase standards), as in many previous reports^2^,^9^. To this end, adequate amounts (1 μL) of liquid standard containing a few ng of target compounds were injected into an ST and purged with pure (99.999%) nitrogen (or helium) gas to evaporate the liquid standard for 5 min at a flow rate of 100–200 mL min^−1^ 9,21. N_2_ gas was used for the purge gas in this experiment with consideration of its cost-efficiency.

In this work, 1 μL of each L-WS (5, 20, and 50 ng μL^−1^) was spiked through the Teflon tubing between the pre-filter and the ST (QW, GW, and QWTN) using a 5 μL syringe (SGE Analytical Science, Australia), while pure N_2_ gas was supplied at flow rates of 0.2 and 1 L min^−1^ (Fig. S4). A pre-filter packed with Carbopack X was utilized to remove possible contaminants present in the N_2_ gas^23^. By adjusting the purge time at each of two N_2_ gas flow rates (5, 50, and 500 min (for 0.2 L min^−1^) and 1, 10, and 100 min (for 1 L min^−1^)), each ST was purged at up to three total volumes (1, 10, and 100 L). To evaluate the relative recovery of STs with different purge parameters, a reference purge method (0.2 L min^−1^ for 3 min) was also employed, as in our previous research^13^. After collecting the vaporized standard of phthalates, each ST was placed in a TD system (TD-20, Shimadzu, Japan) and vaporized at 320 °C for 10 min. These vaporized gas samples were pre-concentrated in a cold trap (combination of QW and Tenax TA) at 5 °C and then thermally desorbed at 320 °C for 10 min. A total of seven target phthalates were then separated on a DB-5MS column (film thickness: 0.25 μm, diameter: 0.25 mm, length: 60 m, Agilent, USA) for detection via GC (GC-2010 Plus, Shimadzu, Japan)/MS (GCMS-QP2010 Ultra, Shimadzu, Japan). The MS system was operated in the electron impact (EI) ionization mode at 70 eV, and ions were scanned in the total ion chromatogram (TIC) mode (range: 40 to 500 m/z) (Table 5). The extracted ion chromatogram (EIC) mode was applied for the quantitation of each compound based on the major mass spectrum described in Table S2. Consequently, the recovery of each phthalate was calculated by dividing the peak area obtained via each purge method into the peak area obtained via the reference purge method.

## Additional Information

**How to cite this article:** Jo, S.-H. *et al*. The combined effects of sampling parameters on the sorbent tube sampling of phthalates in air. *Sci. Rep.* **7**, 45677; doi: 10.1038/srep45677 (2017).

**Publisher's note:** Springer Nature remains neutral with regard to jurisdictional claims in published maps and institutional affiliations.

## Supplementary Material

Supplementary Information

## Figures and Tables

**Table 1:** Comparison of recovery (%) for seven phthalates obtained using quartz wool (QW) tube in relation to concentration levels of standard, purge flow rate, and purge volume.

Concentration level of	N_2_ purge			Recovery (%)^b^						
L-WS^a^ (ng μL^−1^)	flow rate (L min^−1^)	time (min)	volume (L)	DMP	DEP	DBP	BBP	DEHA	DEHP	DOP
5 (Low)	0	0	0	73.0	82.5	84.5	78.3	83.9	77.4	80.9
		5	1	89.6	89.3	95.1	88.6	89.8	91.5	84.9
	0.2	50	10	92.4	93.8	95.9	93.3	93.5	104	93.9
		500	100	93.3	97.2	97.1	92.1	91.6	106	84.4
			Mean	91.8	93.4	96.0	91.3	91.6	100	87.8
			SD	1.90	3.94	1.01	2.49	1.87	7.76	5.35
		1	1	90.2	95.8	93.3	83.4	86.1	92.0	79.8
	1	10	10	94.7	100	94.0	94.3	91.0	81.5	89.9
		100	100	96.0	105	98.6	94.9	92.7	85.9	90.9
			Mean	93.6	100	95.3	90.9	89.9	86.5	86.9
			SD	3.05	4.46	2.90	6.45	3.41	5.27	6.16
20 (Moderate)	0	0	0	91.2	94.1	94.5	90.3	88.3	92.7	85.9
		5	1	95.3	96.0	91.5	92.7	91.4	91.3	86.7
	0.2	50	10	96.3	94.1	88.0	87.1	88.3	106	88.1
		500	100	95.2	93.9	90.3	90.9	92.5	99.4	90.6
			Mean	95.6	94.7	89.9	90.2	90.7	98.7	88.5
			SD	0.62	1.16	1.82	2.87	2.18	7.14	1.99
		1	1	94.2	94.8	88.1	91.3	90.0	90.2	88.4
	1	10	10	94.8	96.8	91.9	97.5	96.2	101	94.1
		100	100	92.1	89.2	86.6	89.5	91.3	86.4	89.8
			Mean	93.7	93.6	88.9	92.8	92.5	92.7	90.8
			SD	1.41	3.96	2.74	4.19	3.28	7.78	2.97
50 (High)	0	0	0	87.9	96.7	93.1	93.1	91.3	90.7	94.1
		5	1	97.2	95.0	96.6	95.3	98.9	96.6	94.6
	0.2	50	10	92.8	90.3	90.7	92.7	95.4	89.8	91.9
		500	100	93.8	89.1	88.3	89.3	91.6	89.1	87.6
			Mean	94.6	91.5	91.9	92.4	95.3	91.8	91.4
			SD	2.29	3.14	4.26	3.01	3.67	4.17	3.55
		1	1	91.7	88.9	92.0	93.4	95.4	91.1	92.6
	1	10	10	91.1	87.5	86.3	88.2	89.6	87.3	87.5
		100	100	91.5	86.8	88.9	89.1	92.5	89.5	89.3
			Mean	91.4	87.7	89.1	90.3	92.5	89.3	89.8
			SD	0.30	1.05	2.82	2.79	2.91	1.93	2.59

^a^Liquid working standard of phthalates.

^b^Recovery = (Peak area obtained via each purge method)/(Peak area obtained via the reference purge method) × 100.

**Table 2:** Comparison of recovery (%) for seven phthalates obtained using glass wool (GW) tube in relation to concentration levels of standard, purge flow rate, and purge volume.

Concentration level of	N_2_ purge			Recovery (%)						
L-WS (ng μL^−1^)	flow rate (L min^−1^)	time (min)	volume (L)	DMP	DEP	DBP	BBP	DEHA	DEHP	DOP
5 (Low)	0	0	0	97.2	100	92.3	59.1	77.8	83.9	70.6
		5	1	81.3	96.4	99.7	97.4	99.5	109	96.6
	0.2	50	10	65.6	85.1	91.7	86.5	90.1	99.0	85.5
		500	100	65.2	71.8	78.3	70.9	84.3	92.3	78.1
			Mean	70.7	84.4	89.9	85.0	91.3	100	86.7
			SD	9.19	12.3	10.8	13.3	7.70	8.39	9.34
		1	1	83.6	98.1	101	98.4	99.7	93.8	98.9
	1	10	10	24.0	66.7	99.9	94.7	95.4	96.4	94.2
		100	100	2.38	29.1	90.3	89.6	94.5	107	90.2
			Mean	36.6	64.6	97.2	94.2	96.5	99.0	94.4
			SD	42.0	34.5	6.07	4.43	2.79	6.95	4.39
20 (Moderate)	0	0	0	108	96.1	95.4	85.6	85.8	87.9	80.5
		5	1	56.7	85.1	99.1	102	101	102	97.8
	0.2	50	10	12.1	52.6	92.5	103	101	102	101
		500	100	14.2	26.0	80.3	103	103	105	106
			Mean	27.7	54.6	90.6	103	102	103	102
			SD	25.2	29.6	9.55	0.62	1.44	1.94	4.09
		1	1	60.1	79.4	94.3	95.2	92.7	102	94.4
	1	10	10	12.7	37.7	87.9	103	99.8	101	100
		100	100	1.05	4.50	46.6	89.0	93.4	98.0	99.4
			Mean	24.6	40.5	76.3	95.6	95.3	100	98.0
			SD	31.3	37.5	25.9	6.79	3.89	2.29	3.08
50 (High)	0	0	0	126	102	92.3	85.2	85.2	94.2	73.6
		5	1	55.7	102	99.9	101	98.0	96.5	97.3
	0.2	50	10	25.3	42.5	89.9	99.2	98.5	92.4	96.9
		500	100	11.1	13.9	56.4	88.7	96.2	96.5	96.4
			Mean	30.7	52.9	82.1	96.2	97.6	95.1	96.9
			SD	22.8	45.1	22.8	6.55	1.18	2.35	0.44
		1	1	61.9	85.4	94.5	97.0	98.2	93.0	95.5
	1	10	10	31.7	42.8	87.5	99.0	96.7	91.9	96.7
		100	100	3.77	3.64	42.9	90.9	96.2	94.7	96.2
			Mean	32.5	44.0	75.0	95.6	97.1	93.2	96.1
			SD	29.1	40.9	28.0	4.21	1.03	1.41	0.61

**Table 3:** Comparison of recovery (%) for seven phthalates obtained using quartz wool plus Tenax TA (QWTN) tube in relation to concentration levels of standard, purge flow rate, and purge volume.

Concentration level of	N_2_ purge			Recovery (%)						
L-WS (ng μL^−1^)	flow rate (L min^−1^)	time (min)	volume (L)	DMP	DEP	DBP	BBP	DEHA	DEHP	DOP
5 (Low)	0	0	0	91.6	95.9	97.6	97.3	93.7	101	93.2
		5	1	107	99.4	102	104	105	98.6	101
	0.2	50	10	99.6	97.4	102	101	99.1	94.1	101
		500	100	104	103	101	101	99.7	97.9	100
			Mean	103	100	102	102	101	96.9	101
			SD	3.70	3.07	0.53	1.96	3.23	2.45	0.50
		1	1	101	100	98.6	99.4	102	97.2	99.4
	1	10	10	96.8	93.3	96.2	96.8	99.0	92.1	94.9
		100	100	97.2	97.1	101	98.3	103	108	97.7
			Mean	98.2	96.8	98.8	98.1	101	99.0	97.3
			SD	2.08	3.47	2.64	1.32	2.09	8.10	2.28
20 (Moderate)	0	0	0	91.8	98.5	94.8	99.5	95.4	99.4	96.7
		5	1	99.7	102	101	103	102	101	96.2
	0.2	50	10	101	109	107	109	105	103	105
		500	100	101	106	107	112	105	108	104
			Mean	101	106	105	108	104	104	102
			SD	1.00	3.20	3.37	4.67	1.58	3.52	5.01
		1	1	102	105	105	104	103	105	101
	1	10	10	91.5	96.3	92.9	93.7	94.5	101	88.8
		100	100	90.4	96.5	88.9	90.6	91.0	102	88.3
			Mean	94.5	99.3	95.7	96.2	96.1	103	92.9
			SD	6.14	5.04	8.54	7.17	5.97	1.81	7.43
50 (High)	0	0	0	94.9	92.9	95.5	92.9	96.2	96.0	96.1
		5	1	102	103	105	108	103	97.4	103
	0.2	50	10	100	108	104	109	102	100	103
		500	100	101	98.9	97.4	93.7	97.0	95.9	95.2
			Mean	101	104	102	104	101	97.7	101
			SD	0.88	4.73	3.98	8.58	3.43	2.09	4.62
		1	1	102	102	98.6	93.8	96.8	101	95.2
	1	10	10	102	98.6	93.9	91.1	96.3	98.9	95.7
		100	100	103	95.8	96.7	94.5	96.9	97.5	96.5
			Mean	102	98.8	96.4	93.1	96.7	99.0	95.8
			SD	0.48	3.01	2.37	1.79	0.35	1.56	0.61

**Table 4:** Results of one-way analysis of variance (ANOVA) test for recovery of phthalates in relation to two contrasting purge flow rates (0.2 vs. 1 L min^−1^).

	Recovery (%) with different purge flow rate (L min^−1^)													
Conc. level of	DMP		DEP		DBP		BBP		DEHA		DEHP		DOP	
L-WS (ng μL^−1^)	0.2	1	0.2	1	0.2	1	0.2	1	0.2	1	0.2	1	0.2	1
*Quartz wool (QW) tube*														
5 (Low)	89.6	90.2	89.3	95.8	95.1	93.3	88.6	83.4	89.8	86.1	91.5	92.0	84.9	79.8
	92.4	94.7	93.8	100	95.9	94.0	93.3	94.3	93.5	91.0	104	81.5	93.9	89.9
	93.3	96.0	97.2	105	97.1	98.6	92.1	94.9	91.6	92.7	106	85.9	84.4	90.9
20 (Moderate)	95.3	94.2	96.0	94.8	91.5	88.1	92.7	91.3	91.4	90.0	91.3	90.2	86.7	88.4
	96.3	94.8	94.1	96.8	88.0	91.9	87.1	97.5	88.3	96.2	106	101	88.1	94.1
	95.2	92.1	93.9	89.2	90.3	86.6	90.9	89.5	92.5	91.3	99.4	86.4	90.6	89.8
50 (High)	97.2	91.7	95.0	88.9	96.6	92.0	95.3	93.4	98.9	95.4	96.6	91.1	94.6	92.6
	92.8	91.1	90.3	87.5	90.7	86.3	92.7	88.2	95.4	89.6	89.8	87.3	91.9	87.5
	93.8	91.5	89.1	86.8	88.3	88.9	89.3	89.1	91.6	92.5	89.1	89.5	87.6	89.3
p-value	0.32		0.79		0.40		0.98		0.55		**0.02**^**a**^		0.98	
*Glass wool (GW) tube*														
5 (Low)	81.3	83.6	96.4	98.1	99.7	101	97.4	98.4	99.5	99.7	109	93.8	96.6	98.9
	65.6	24.0	85.1	66.7	91.7	99.9	86.5	94.7	90.1	95.4	99.0	96.4	85.5	94.2
	65.2	2.38	71.8	29.1	78.3	90.3	70.9	89.6	84.3	94.5	92.3	107	78.1	90.2
20 (Moderate)	56.7	60.1	85.1	79.4	99.1	94.3	102	95.2	101	92.7	102	102	97.8	94.4
	12.1	12.7	52.6	37.7	92.5	87.9	103	103	101	99.8	102	101	101	100
	14.2	1.05	26.0	4.50	80.3	46.6	103	89.0	103	93.4	105	98.0	106	99.4
50 (High)	55.7	61.9	102	85.4	99.9	94.5	101	97.0	98.0	98.2	96.5	93.0	97.3	95.5
	25.3	31.7	42.5	42.8	89.9	87.5	99.2	99.0	98.5	96.7	92.4	91.9	96.9	96.7
	11.1	3.77	13.9	3.64	56.4	42.9	88.7	90.9	96.2	96.2	96.5	94.7	96.4	96.2
p-value	0.40		0.37		0.60		0.89		0.80		0.48		0.72	
*Quartz wool plus Tenax TA (QWTN) tube*														
5 (Low)	107	101	99.4	100	102	98.6	104	99.4	105	102	98.6	97.2	101	99.4
	99.6	96.8	97.4	93.3	102	96.2	101	96.8	99.1	99.0	94.1	92.1	101	94.9
	104	97.2	103	97.1	101	101	101	98.3	99.7	103	97.9	108	100	97.7
20 (Moderate)	99.7	102	102	105	101	105	103	104	102	103	101	105	96.2	101
	101	91.5	109	96.3	107	92.9	109	93.7	105	94.5	103	101	105	88.8
	101	90.4	106	96.5	107	88.9	112	90.6	105	91.0	108	102	104	88.3
50 (High)	102	102	103	102	105	98.6	108	93.8	103	96.8	97.4	101	103	95.2
	100	102	108	98.6	104	93.9	109	91.1	102	96.3	100	98.9	103	95.7
	101	103	98.9	95.8	97.4	96.7	93.7	94.5	97.0	96.9	95.9	97.5	95.2	96.5
p-value	0.05		**0.02**		**0.01**		**0.002**		**0.03**		0.78		**0.01**	

^a^The p-value in bold type is significant at 0.05 level.

**Table 5:** Operational conditions of a thermal desorber (TD)-gas chromatography (GC)/mass spectrometry (MS) system for the analysis of phthalates.

(*A) Thermal desorption (TD) system (TD-20, Shimadzu, Japan*)		
Sampling tube: QW^a^, GW^b^, or QW(TN)^c^ in a quartz tube (6.35 mm × 90 mm)		
Carrier gas:	He (99.999%)	
Desorption flow:	100	mL min^−1^
Desorption temp:	320	°C
Desorption time:	10	min
Cold trap: QW 10 mg and Tenax TA 50 mg (3.2 mm outer diameter × 100 mm)		
Carrier gas:	He (99.999%)	
Transfer line temp:	300	°C
Adsorption temp:	10	°C
Desorption temp:	320	°C
Desorption time:	10	min
Column flow:	1.8 (constant)	mL min^−1^
Split ratio:	5	
Purge gas flow:	3	mL min^−1^
(*B) Gas chromatography (GC-2010 Plus, Shimadzu, Japan*)		
Column: DB-5MS (Agilent J&W, USA), Length: 60 m, Film thickness: 0.25 μm, Diameter: 0.25 mm		
Initial temp:	80	°C
Initial hold:	5	min
Oven rate:	20	°C min^−1^
Final temp:	300	°C
Final hold:	24	min
Total analysis time:	40	min
(*C) Mass spectrometry (GCMS-QP2010 Ultra, Shimadzu, Japan*)		
Ionization mode:	EI (70 eV)	
Ion source temp:	280	°C
Interface temp:	280	°C
TIC scan range:	40–500	m z^−1^

^a^Quartz wool (only 10 mg of QW was packed in each quartz tube).

^b^Glass wool (only 10 mg of GW was packed in each quartz tube).

^c^Combination of Quartz wool and Tenax TA (Quartz wool 10 mg + Tenax TA 50 mg in each quartz tube).
