# Supplementary material for: The combined effects of sampling parameters on the sorbent tube sampling of phthalates in air
Source: Sci Rep. 2017 Mar 31;7:45677. doi: 10.1038/srep45677 (PMC5374508; doi:10.1038/srep45677)
Supplement: Supplementary Information [file srep45677-s1.doc]

**Supplementary Information**

**The combined effects of sampling parameters on the sorbent tube sampling of phthalates in air**

Sang-Hee Jo^1^, Ki-Hyun Kim^1,*^, Kyenghee Kwon^2^

^1^Department of Civil and Environmental Engineering, Hanyang University, 222 Wangsimni-ro, Seoul, 04763, Korea

^2^College of Pharmacy, Dongkuk University, Goyang-si 10326, Korea

*Corresponding author: kkim61@hanyang.ac.kr, 82-2-2220-2325 (Tel), 82-2-2220-1945 (Fax)

**Supplementary Figure Legends**

**Figure S1. A schematic diagram for the collection of the vaporized liquid standard of phthalates by employing an N_2_ purge method.**

**Figure S2. Recovery (%) of all target phthalates with various types of sorbent tube as a function of purge volume (up to 100 L).**

**Figure S3. Recovery (%) of phthalate concentrations for each sorbent tube type across phthalates at four purge volumes (0, 1, 10, and 100 L).**

**Figure S4. Comparison of average recovery (%) based on purge flow rate (0.2 and 1 L min^-1^).**

Table S1. Comparison of calibration analysis and quality assurance (QA) results between three sorbent tube (ST) types acquired via a thermal desorption (TD)-gas chromatography (GC)/mass spectrometry (MS) system.

| Order | Compound | Quartz wool (QW) tube | | | | |  | Glass wool (GW) tube | | | | |  | Quartz wool plus Tenax TA (QWTN) tube | | | | |
| --- | --- | --- | --- | --- | --- | --- | --- | --- | --- | --- | --- | --- | --- | --- | --- | --- | --- | --- |
|  |  | RF^a]^ | R^2^ | RSD (%)^b]^ | MDL (pg)^c]^ | MDL (ng m^-3^)^d]^ |  | RF | R^2^ | RSD (%) | MDL (pg) | MDL (ng m^-3^) |  | RF | R^2^ | RSD (%) | MDL (pg) | MDL (ng m^-3^) |
| 1 | DMP | 27,716 | 0.9940 | 2.02 | 48.5 | 0.48 |  | 16,611 | 0.9945 | 7.47 | 57.6 | 0.58 |  | 24,660 | 0.9906 | 0.55 | 56.4 | 0.56 |
| 2 | DEP | 31,582 | 0.9963 | 1.00 | 17.8 | 0.18 |  | 24,191 | 0.9998 | 8.42 | 46.5 | 0.47 |  | 28,821 | 0.9978 | 0.60 | 77.9 | 0.78 |
| 3 | DBP | 50,586 | 0.9911 | 0.37 | 65.9 | 0.66 |  | 44,954 | 0.9991 | 2.02 | 60.4 | 0.60 |  | 45,894 | 0.9915 | 1.88 | 53.9 | 0.54 |
| 4 | BBP | 21,130 | 0.9963 | 1.55 | 31.8 | 0.32 |  | 18,675 | 0.9961 | 0.27 | 44.3 | 0.44 |  | 19,578 | 0.9978 | 1.74 | 47.3 | 0.47 |
| 5 | DEHA | 25,803 | 0.9940 | 1.94 | 36.2 | 0.36 |  | 22,937 | 0.9991 | 1.64 | 21.4 | 0.21 |  | 23,279 | 0.9946 | 2.75 | 76.8 | 0.77 |
| 6 | DEHP | 33,871 | 0.9951 | 2.83 | 400 | 4.00 |  | 29,352 | 0.9917 | 6.83 | 627 | 6.27 |  | 29,516 | 0.9952 | 1.56 | 1,620 | 16.2 |
| 7 | DOP | 58,010 | 0.9945 | 2.30 | 39.2 | 0.39 |  | 50,117 | 0.9973 | 0.50 | 32.9 | 0.33 |  | 51,890 | 0.9931 | 4.01 | 50.0 | 0.50 |

^a]^Response factor value = (Peak area of each compound) / (Injected mass amount)

^b]^Relative standard deviation (10 ng μL^-1^ of L-WS was used to measure RSD values)

^c]^Method detection limit

^d]^Assuming that sampling volume is 100 L

Table S2. Information regarding the basic properties of the seven target phthalate compounds selected in this study

| Order | Compound | Short name | Formula | CAS  number | Molecular weight | Boiling point | Mass spectrum^a]^ | Retention time |
| --- | --- | --- | --- | --- | --- | --- | --- | --- |
|  |  |  |  |  | (g mol^-1^) | (℃) | (m z^-1^) | (min) |
| 1 | Dimethyl phthalate | DMP | C_10_H_10_O_4_ | 131-11-3 | 194 | 284 | 163 | 13.0 |
| 2 | Diethyl Phthalate | DEP | C_12_H_14_O_4_ | 84-66-2 | 222 | 295 | 149 | 13.9 |
| 3 | Dibutyl phthalate | DBP | C_16_H_22_O_4_ | 84-74-2 | 278 | 340 | 149 | 16.0 |
| 4 | Benzyl butyl phthalate | BBP | C_19_H_20_O_4_ | 85-68-7 | 312 | 370 | 149 | 18.1 |
| 5 | Di(2-ethylhexyl) adipate | DEHA | C_22_H_42_O_4_ | 103-23-1 | 371 | 417 | 129 | 18.2 |
| 6 | Di(2-ethylhexyl) phthalate | DEHP | C_24_H_38_O_4_ | 117-81-7 | 391 | 385 | 149 | 19.2 |
| 7 | Di-n-octyl phthalate | DOP | C_24_H_38_O_4_ | 117-84-0 | 391 | 380 | 149 | 20.9 |

^a]^Major mass spectrum was used for the quantitation of each compound

(A) Dimethyl phthalate (DMP)


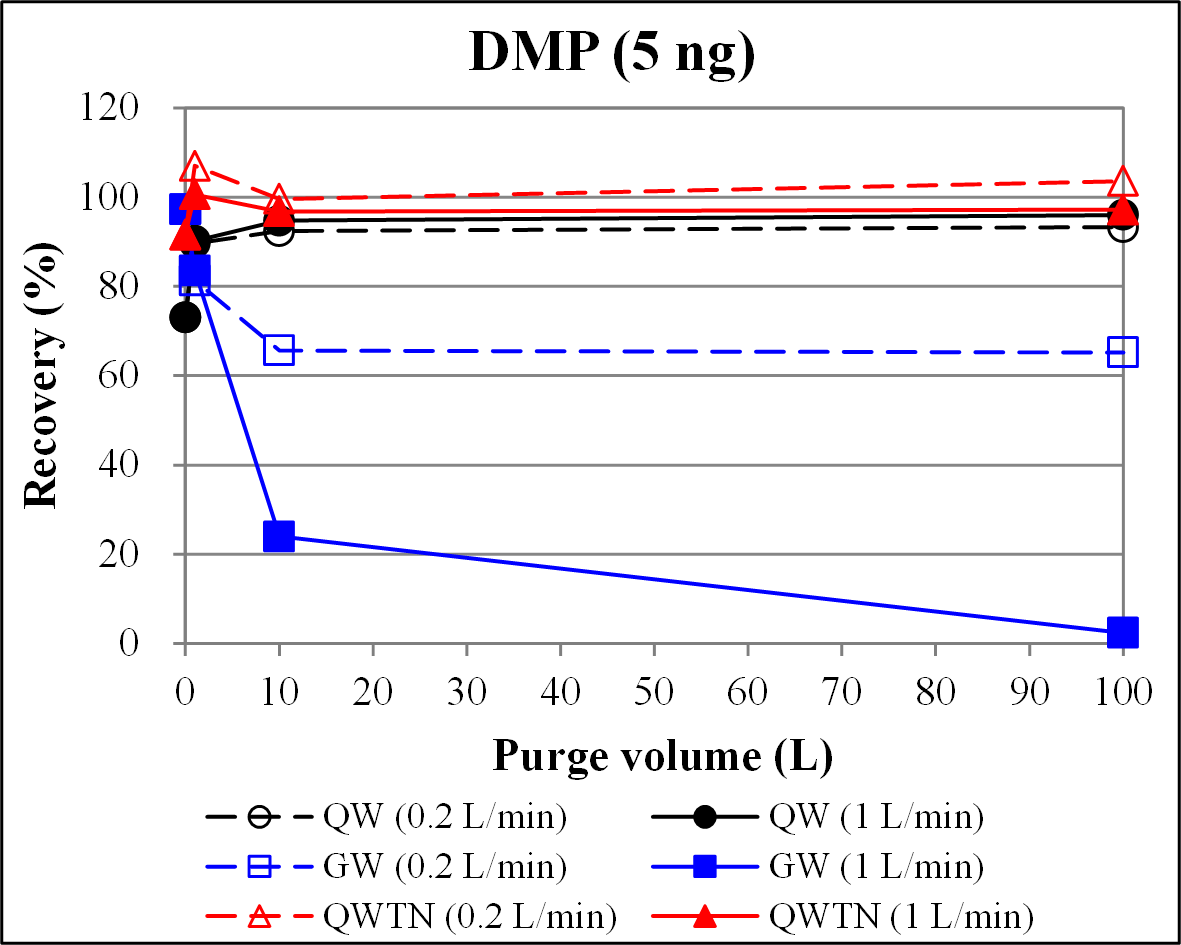

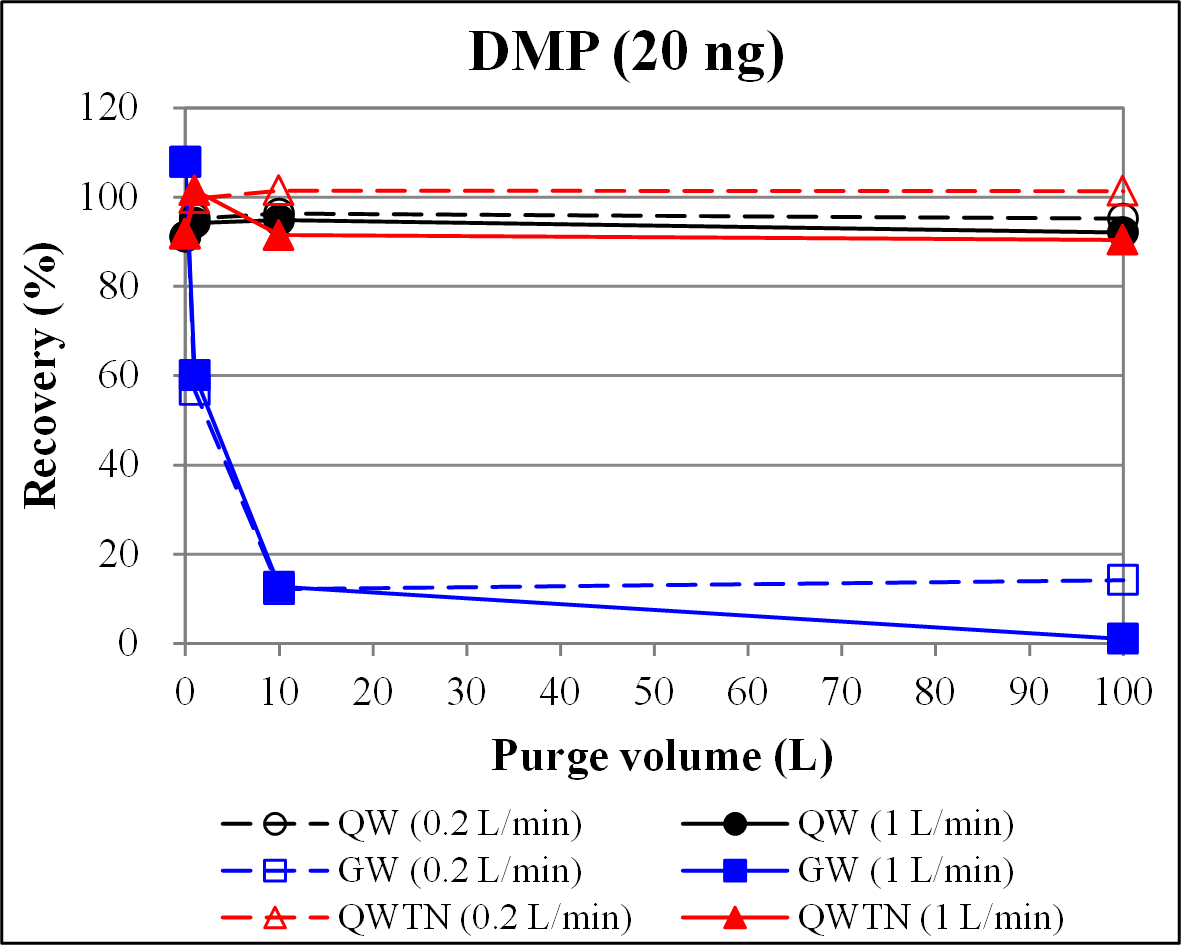

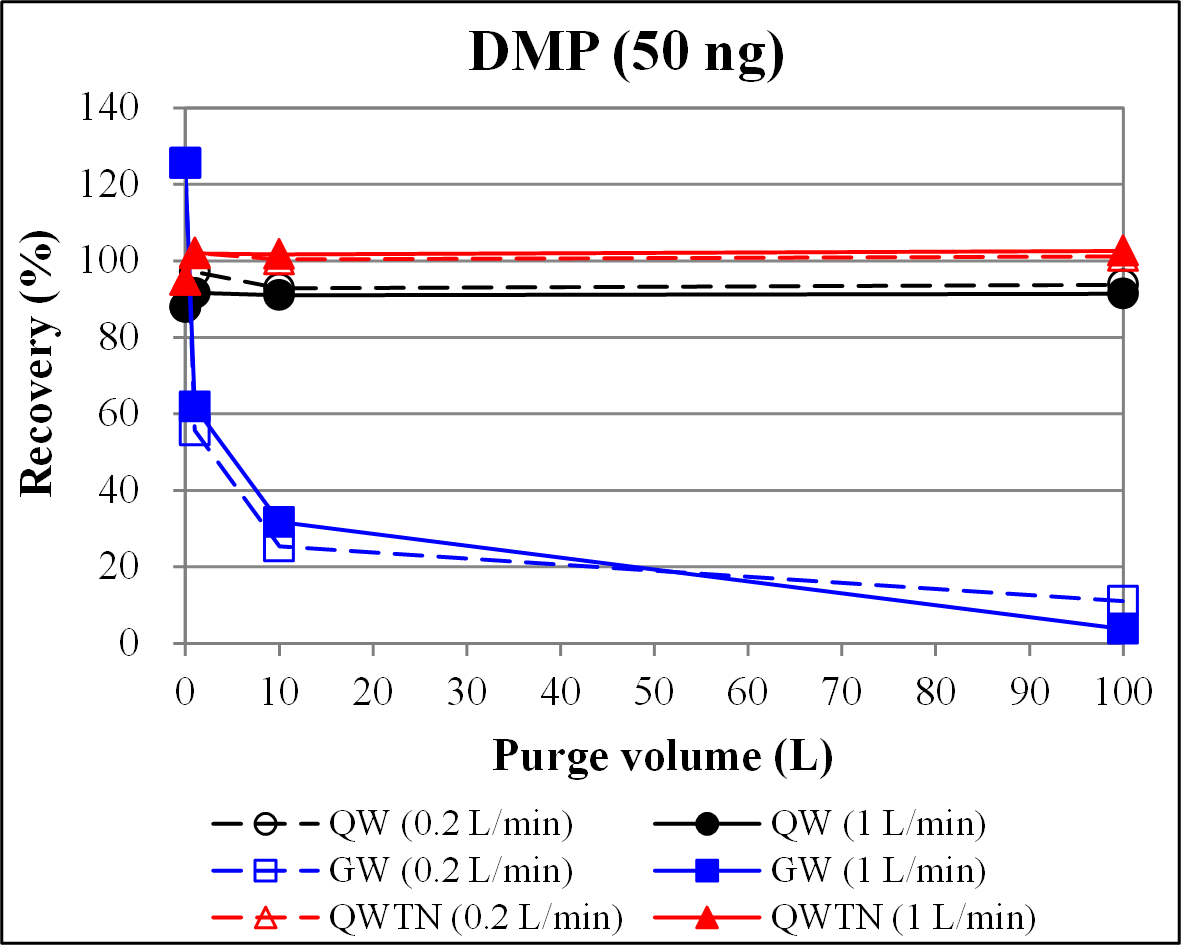


(B) Diethyl phthalate (DEP)


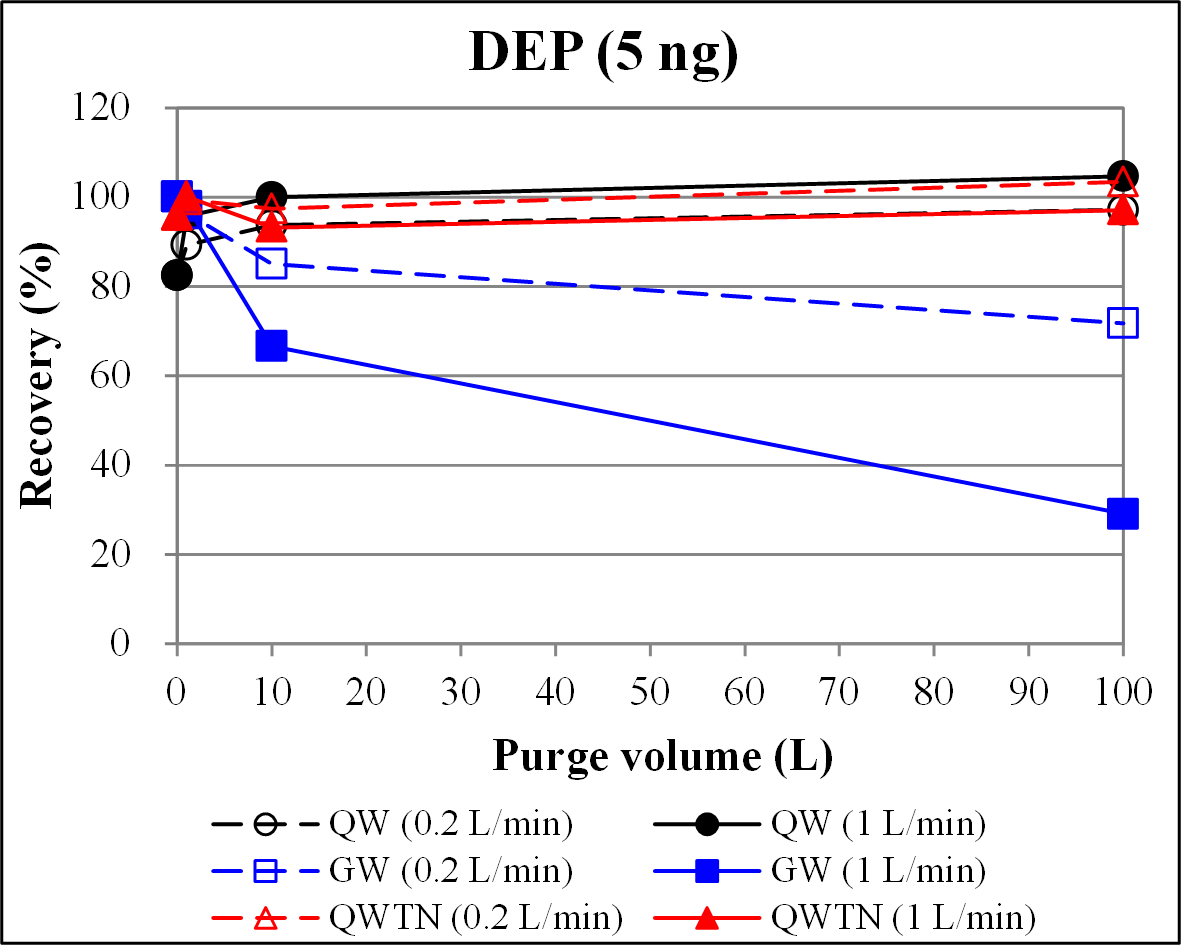

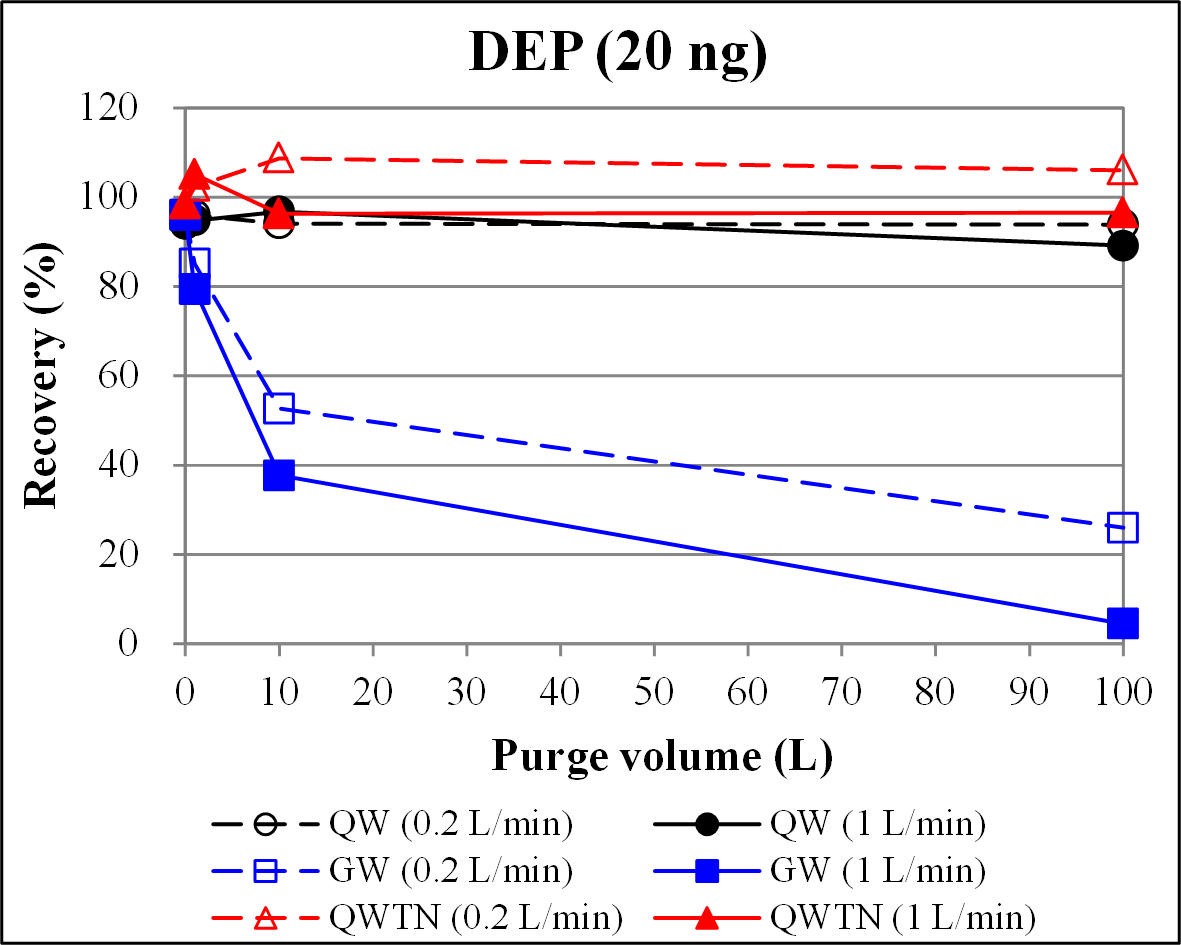

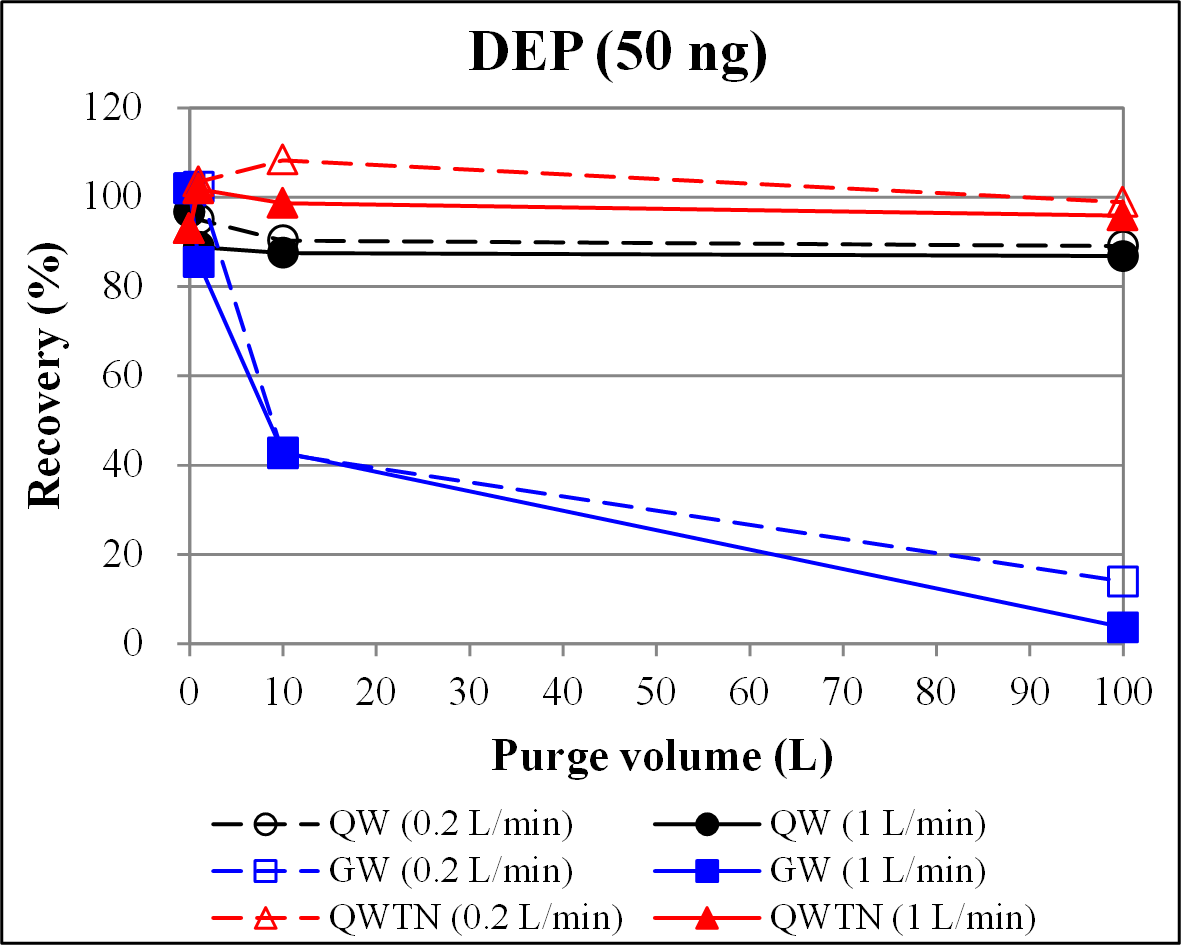


(C) Dibutyl phthalate (DBP)


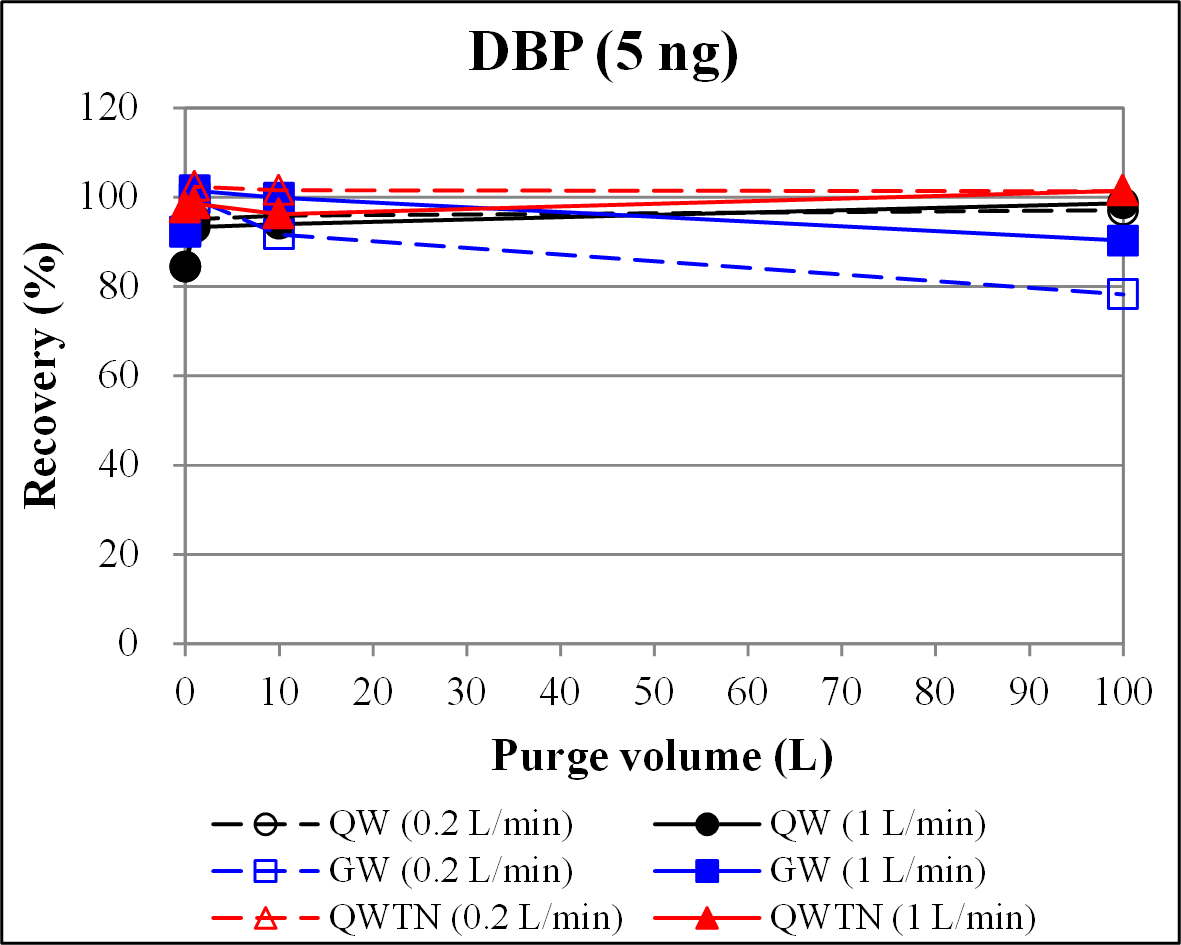

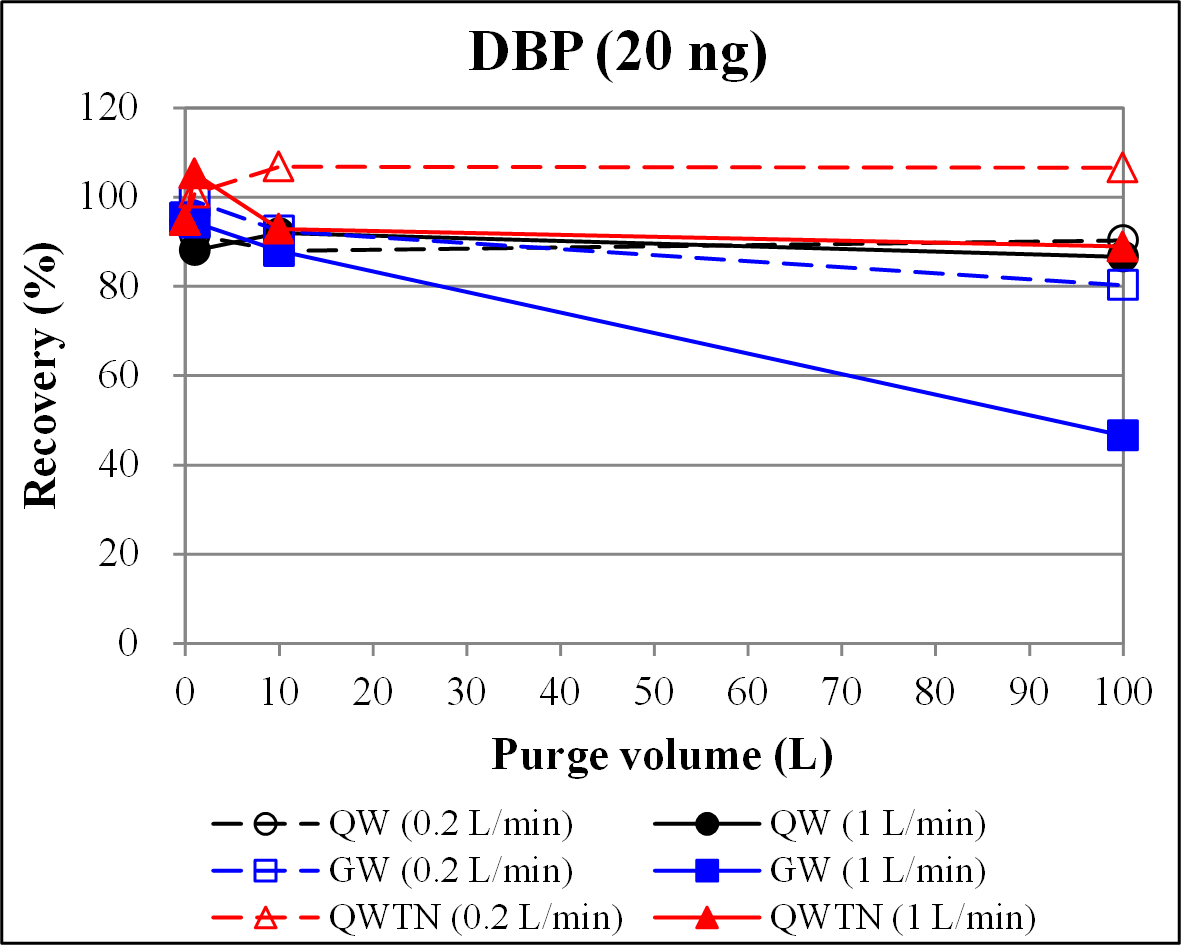

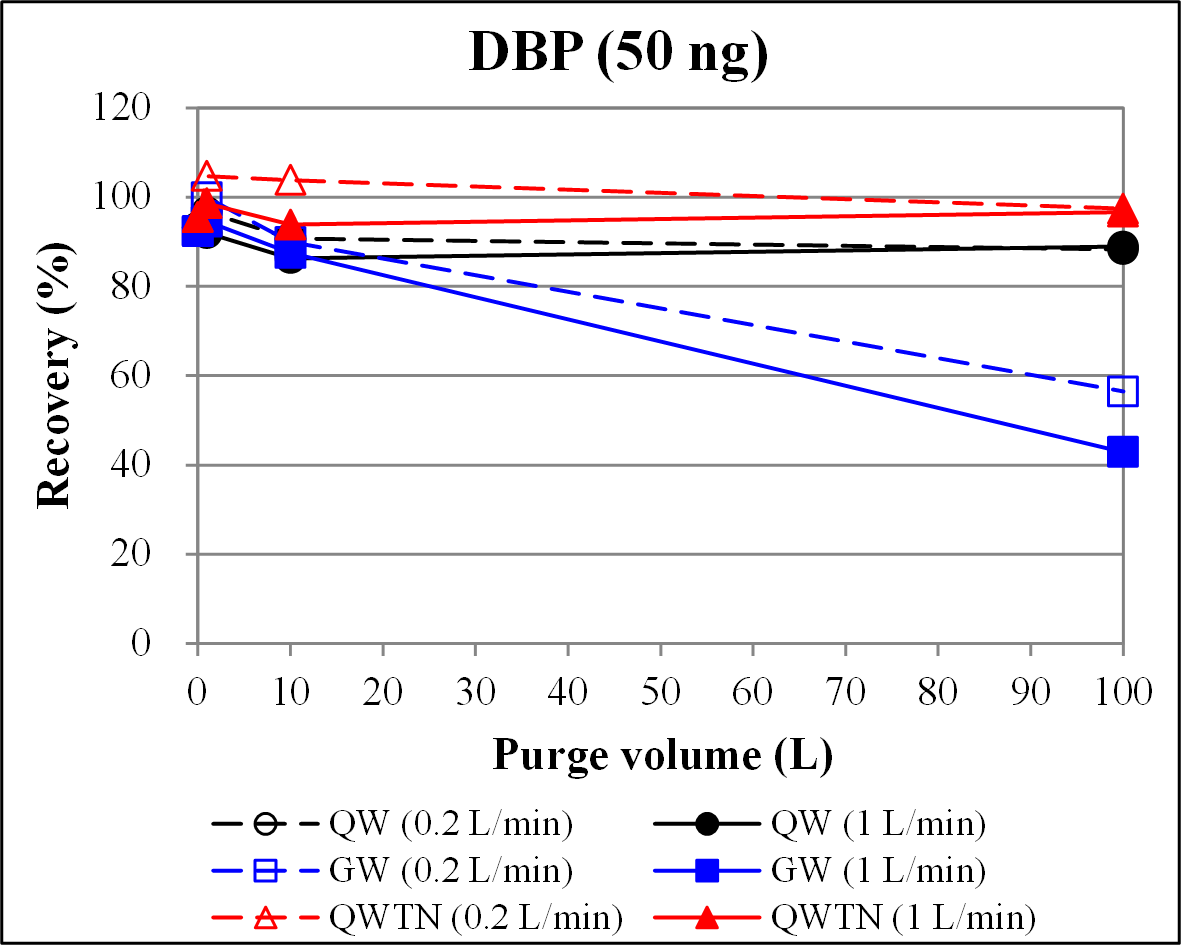


(D) Benzyl butyl phthalate (BBP)


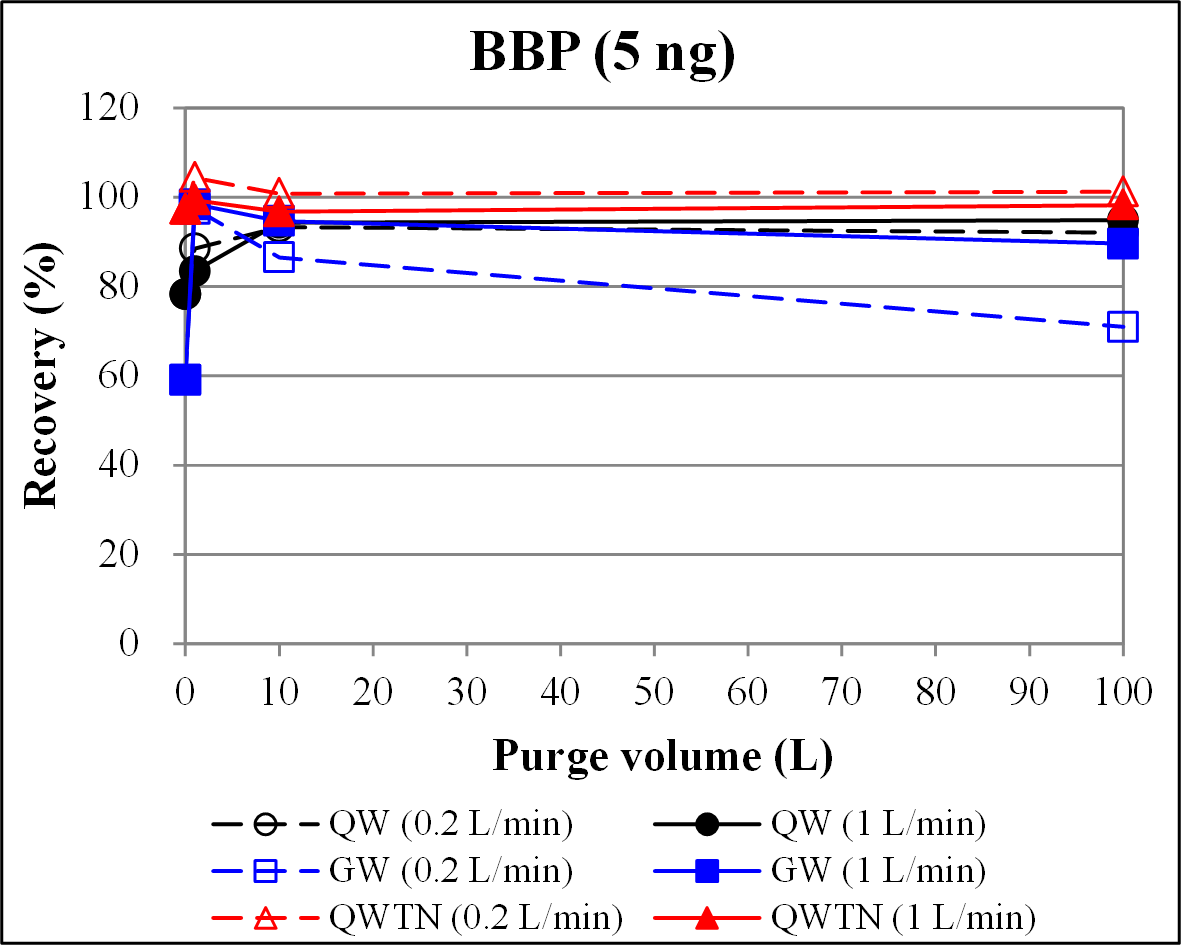

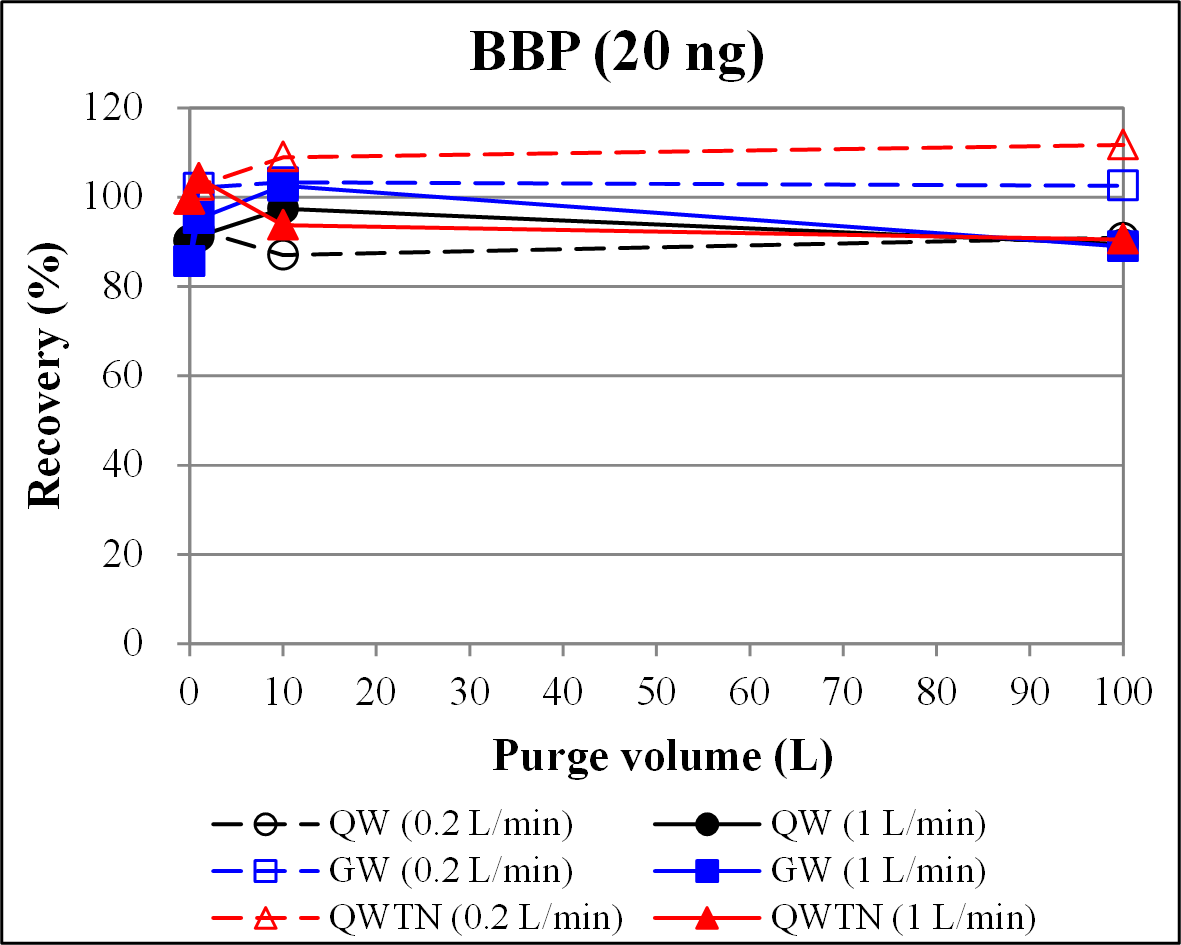

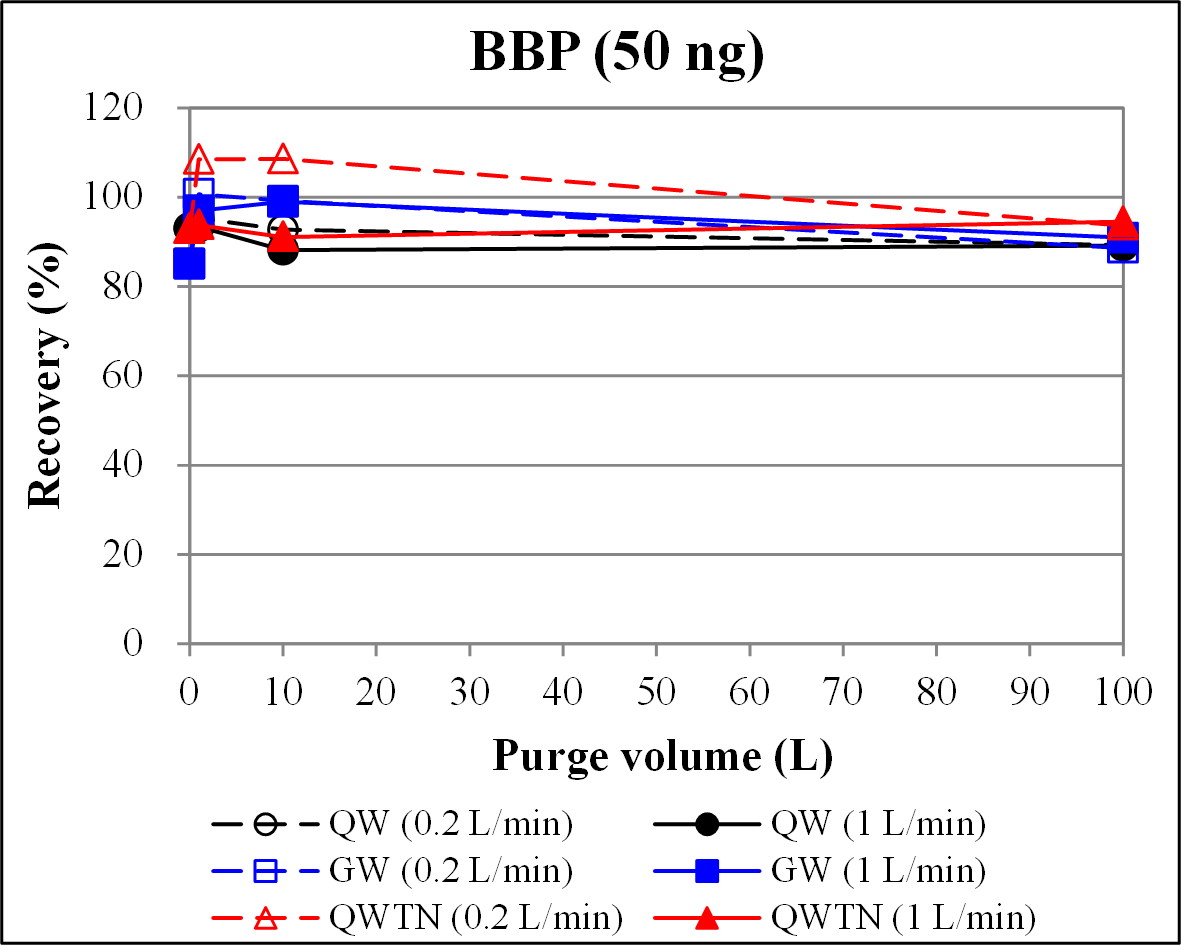


(E) Di(2-ethylhexyl) adipate (DEHA)


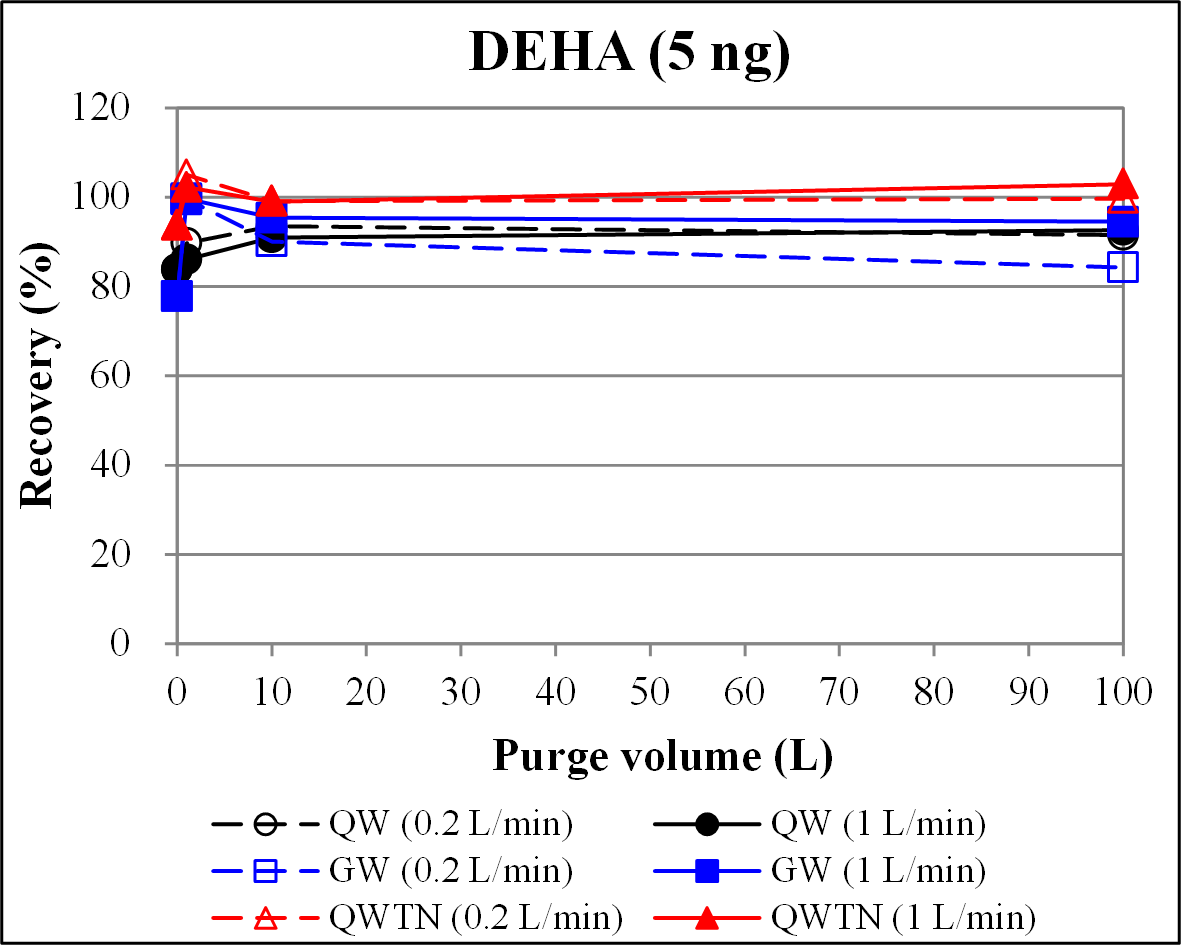

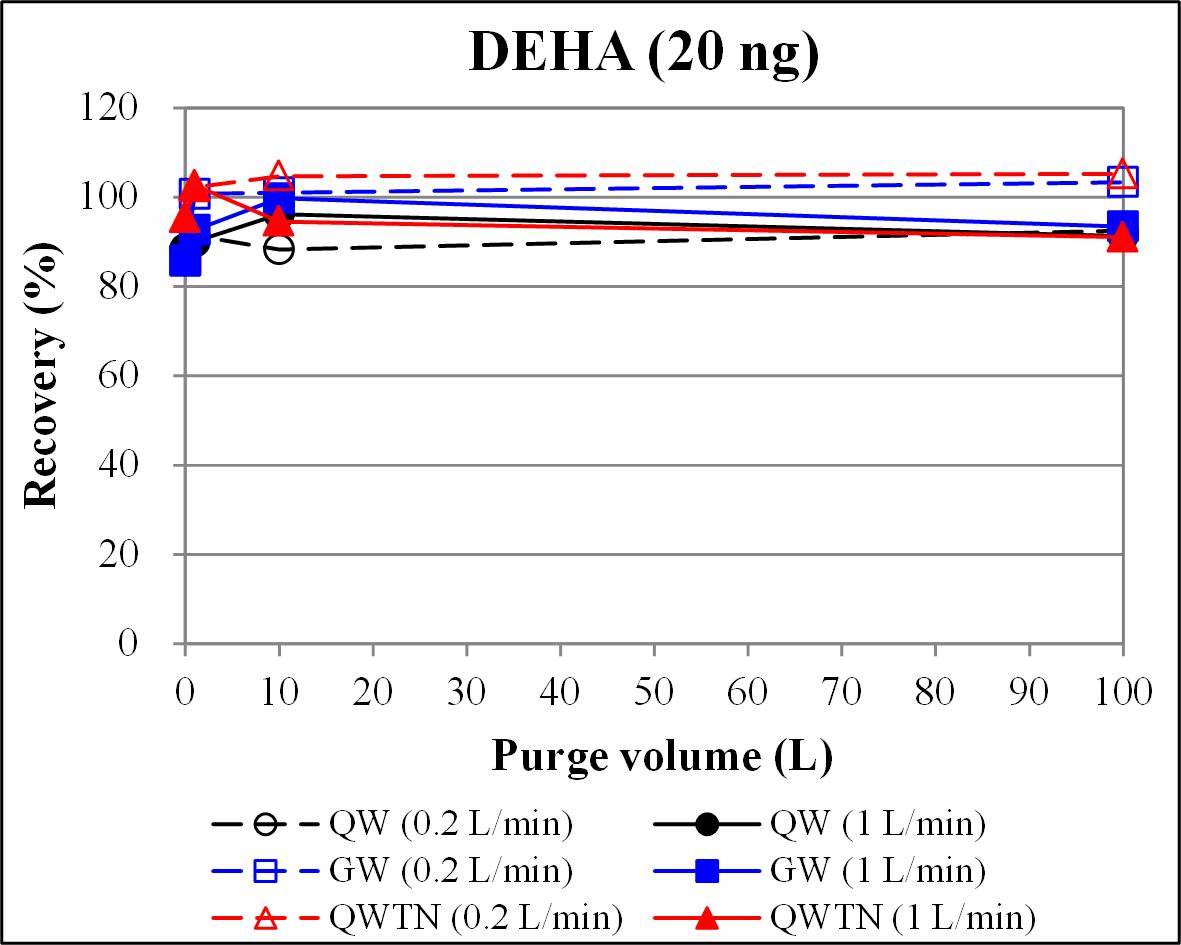

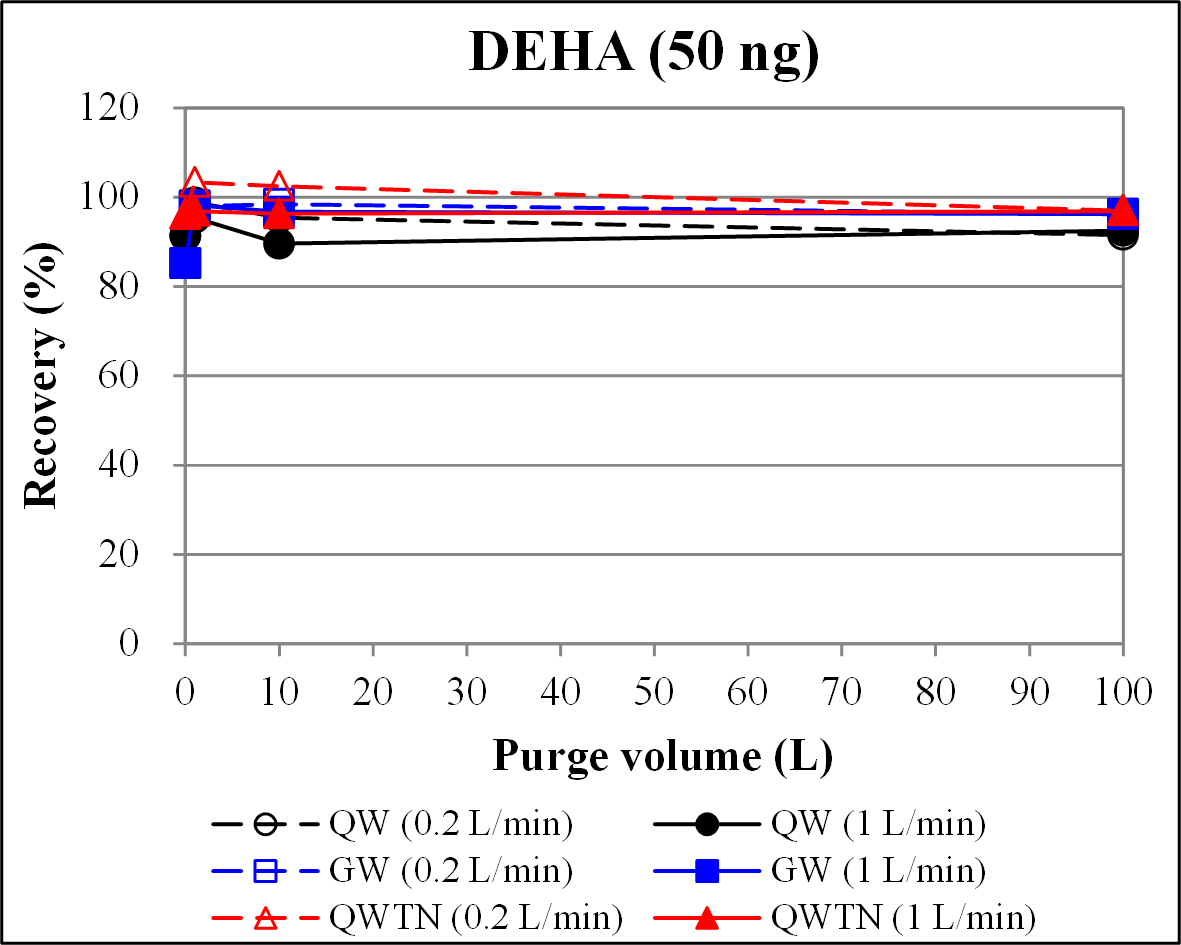


(F) Di(2-ethylhexyl) phthalate (DEHP)


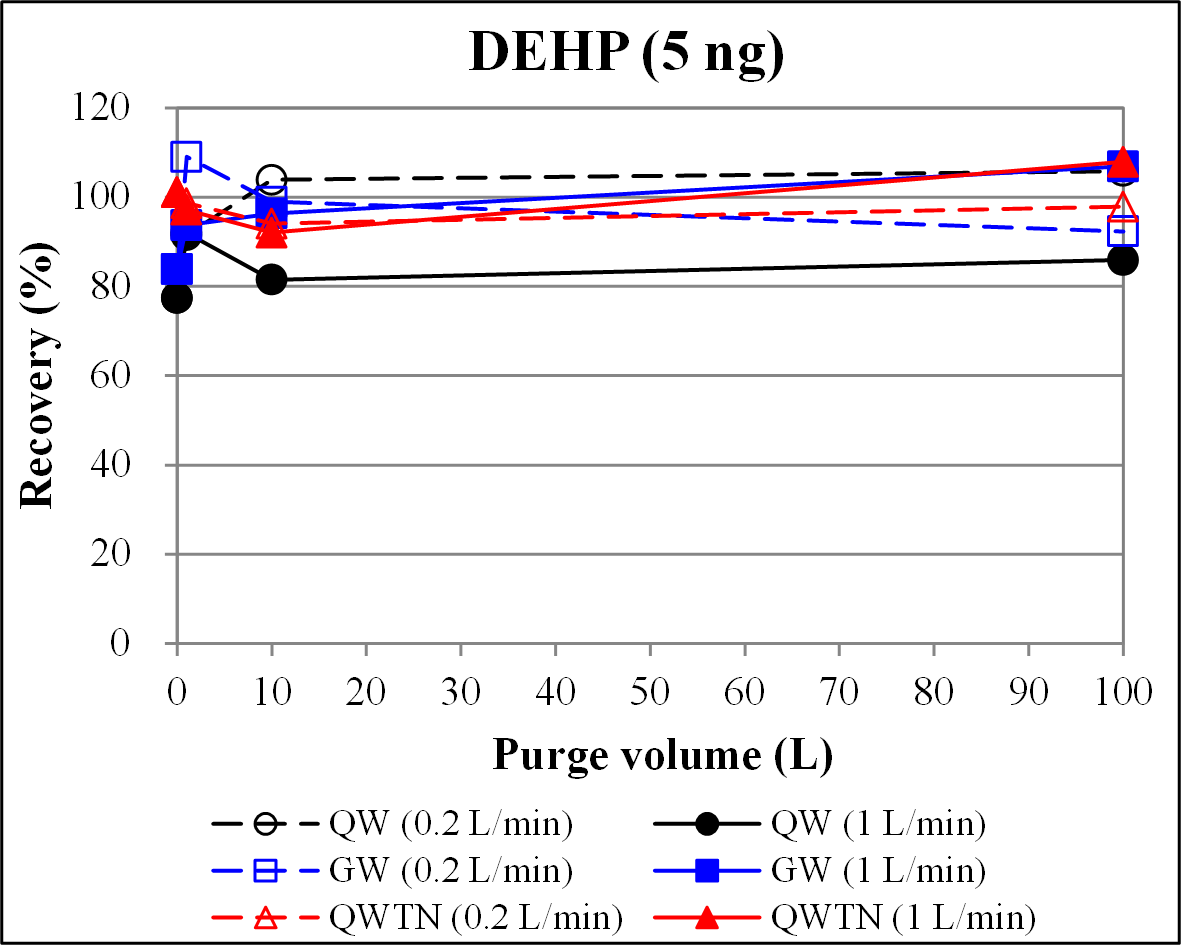

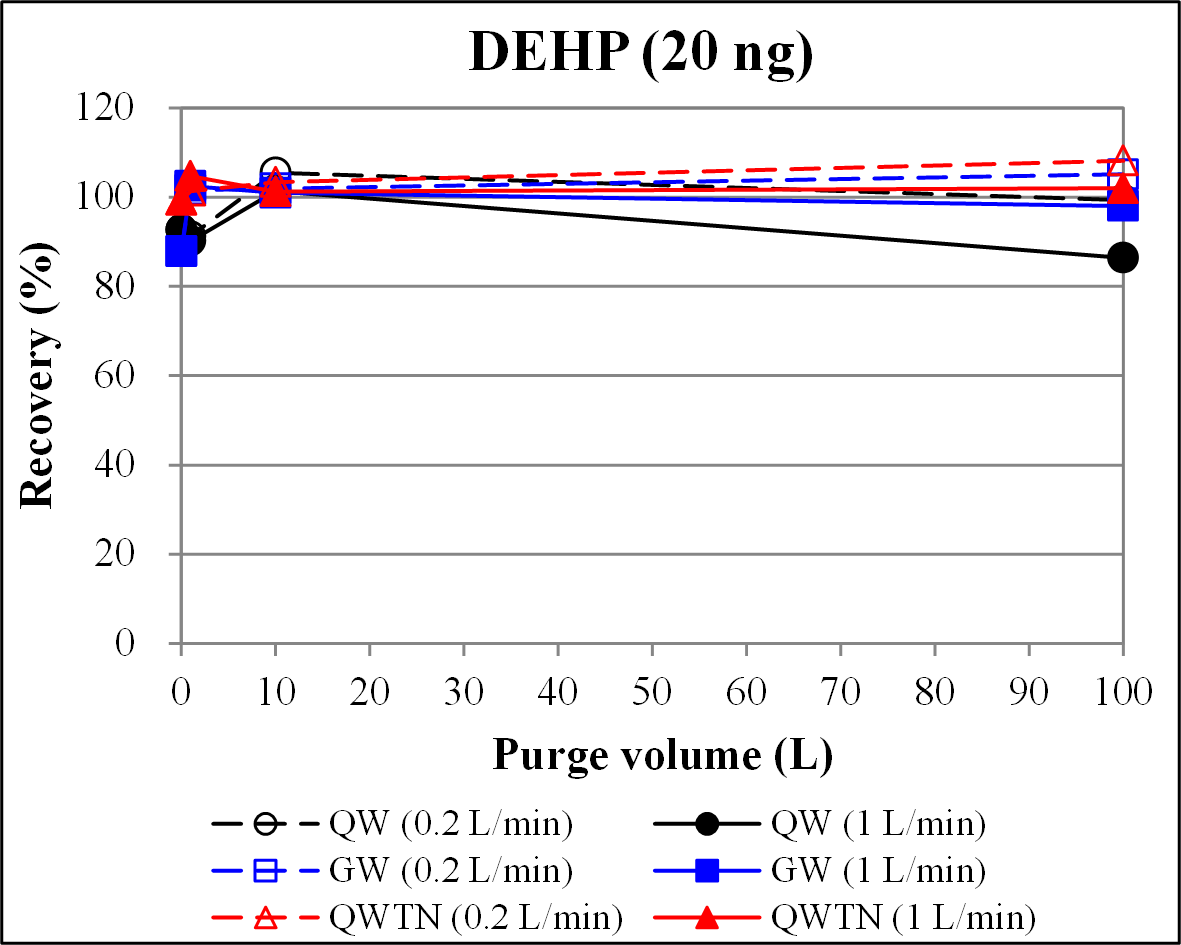

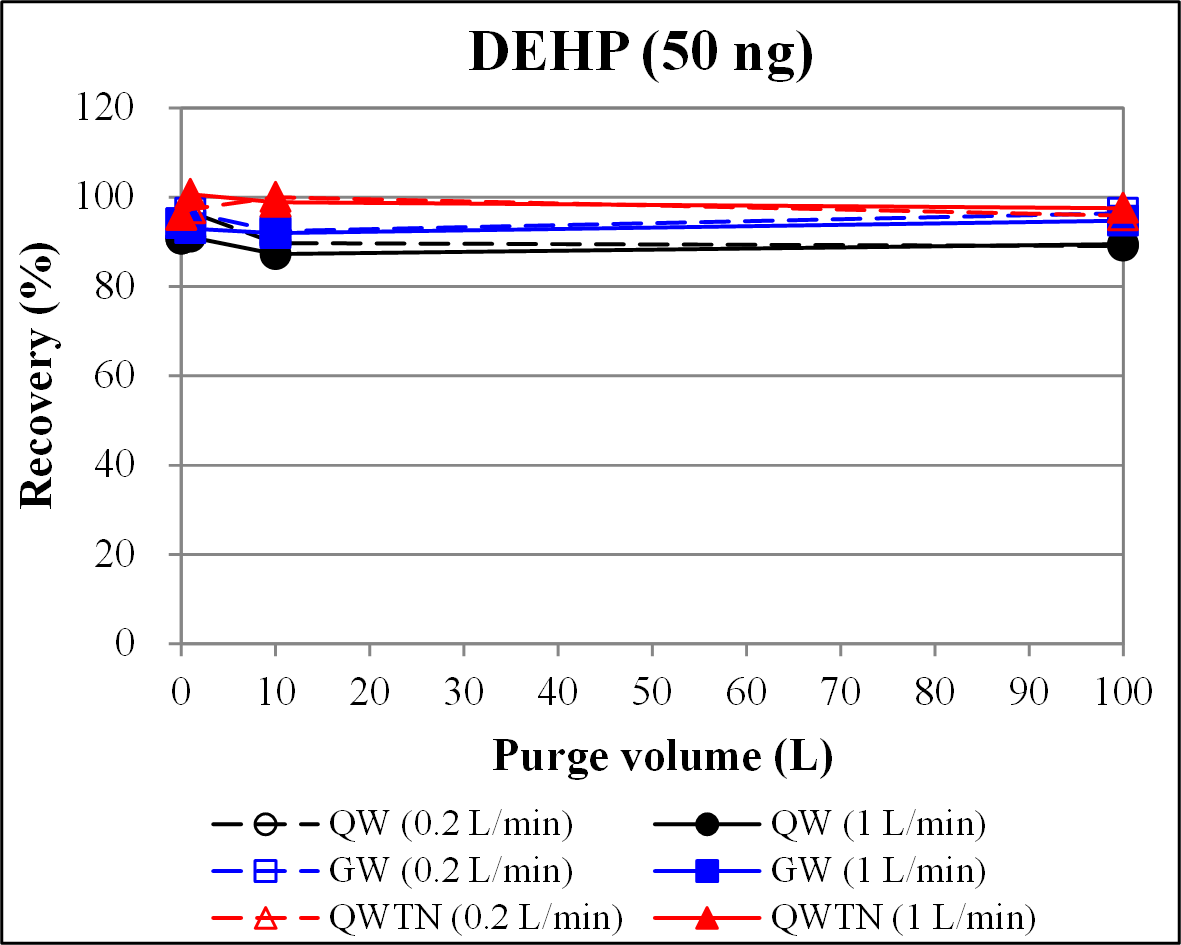


(G) Di-n-octyl phthalate (DOP)


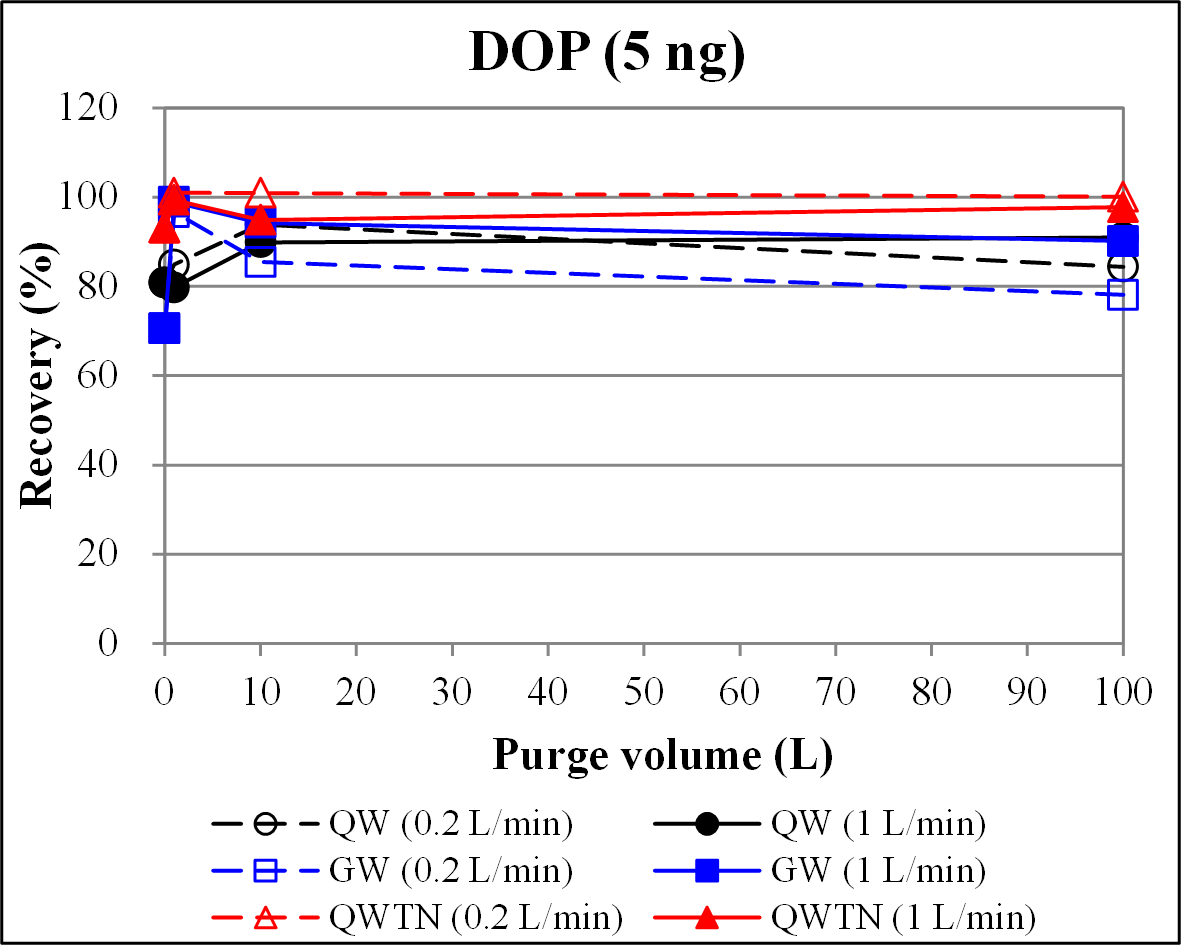

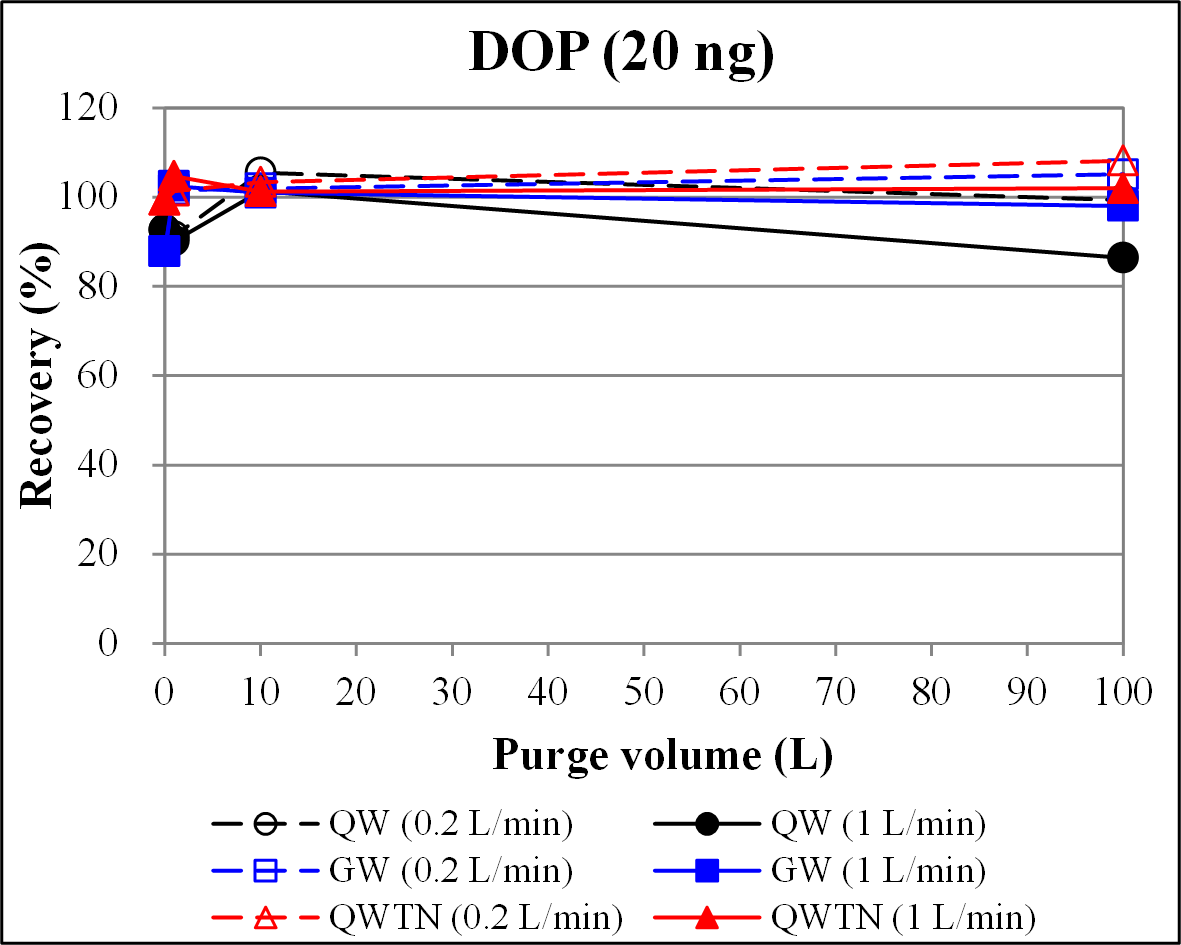

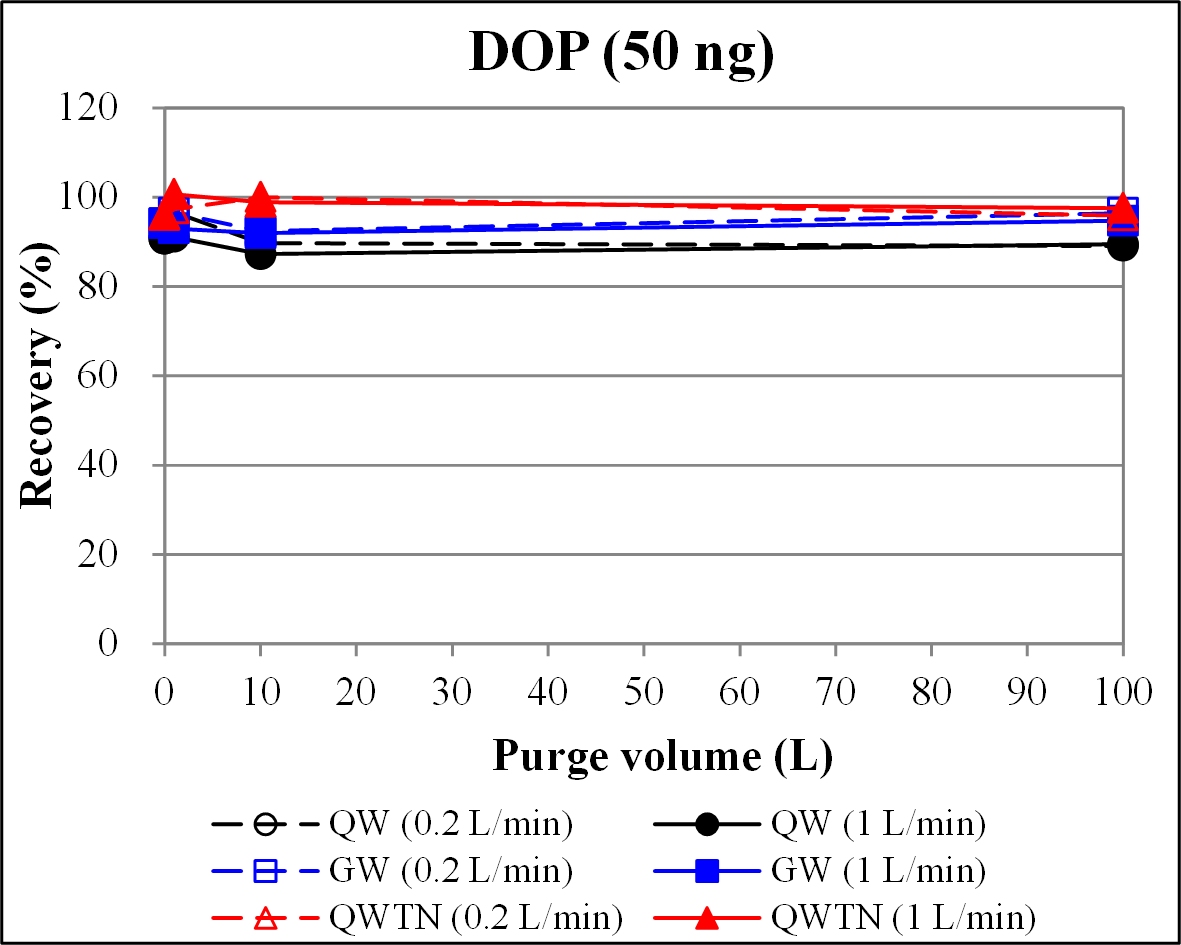


Figure S1. Recovery (%) of all target phthalates with various types of sorbent tube as a function of purge volume (up to 100 L).

(A) Quartz wool (QW) tube


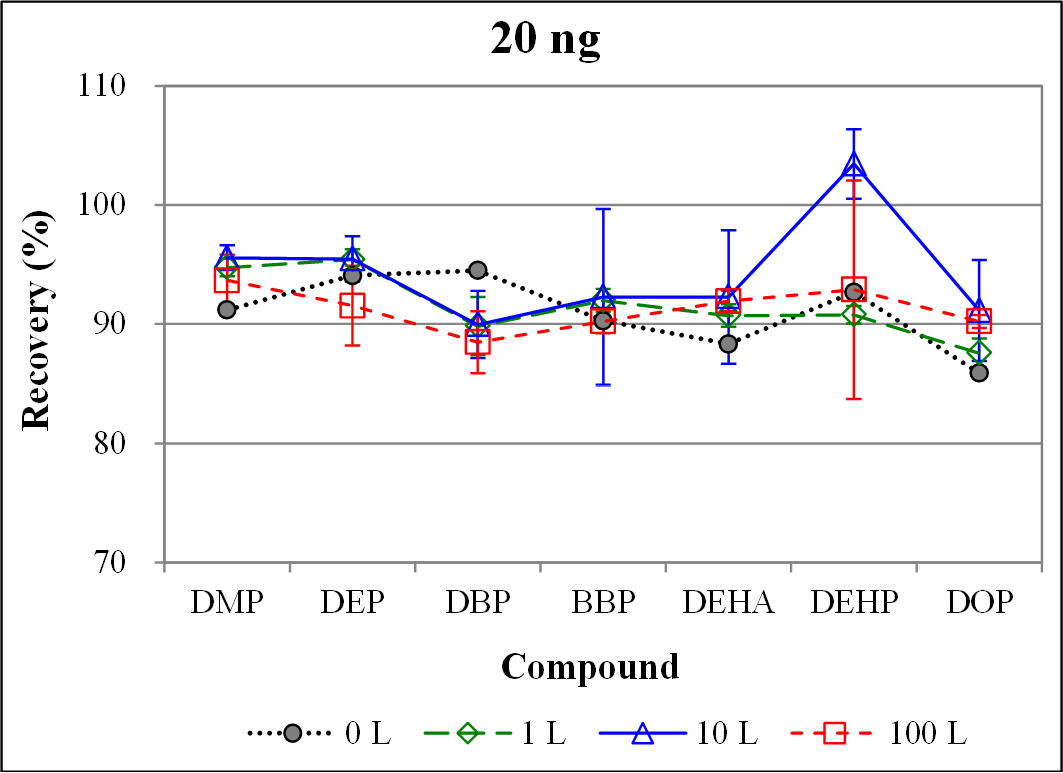


(B) Glass wool (GW) tube


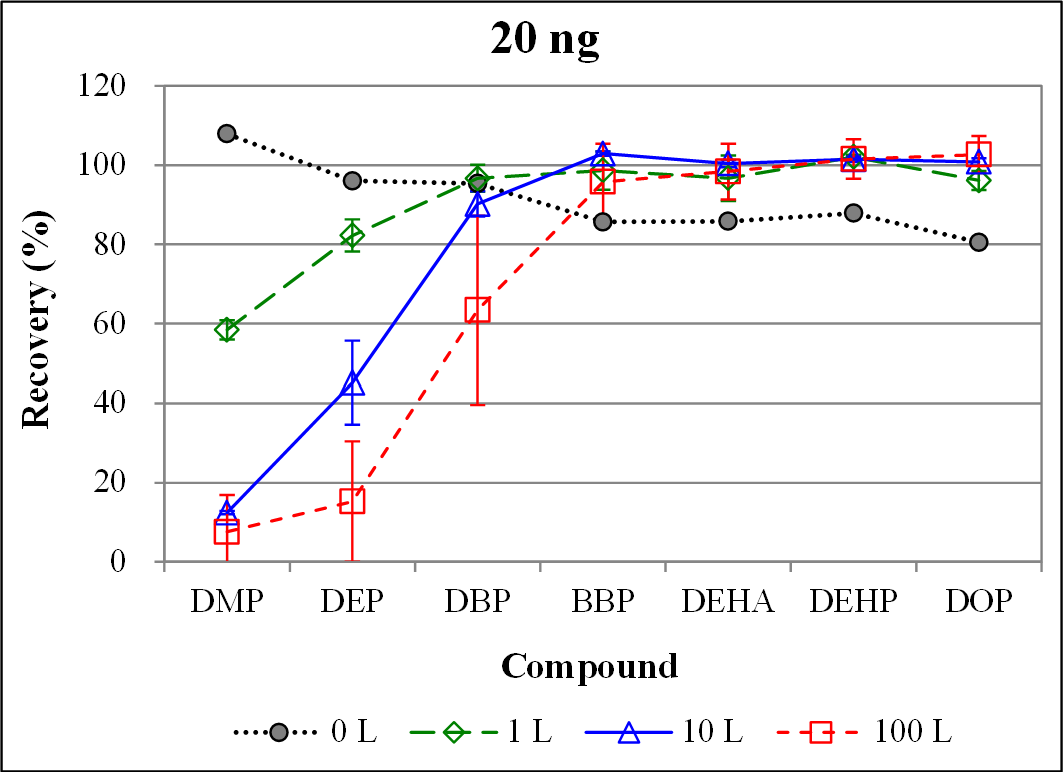


(C) Quartz wool plus Tenax TA (QWTN) tube


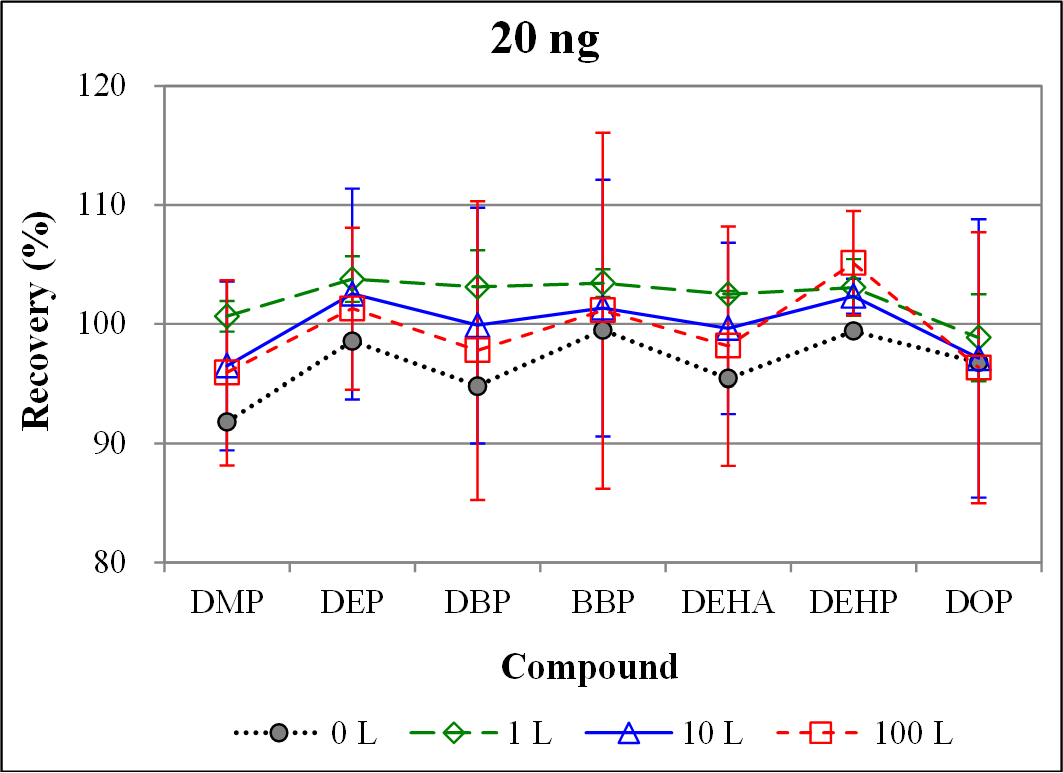


Figure S2. Recovery (%) of phthalate concentrations for each sorbent tube type across phthalates at four purge volumes (0, 1, 10, and 100 L).

(A) Quartz wool (QW) tube


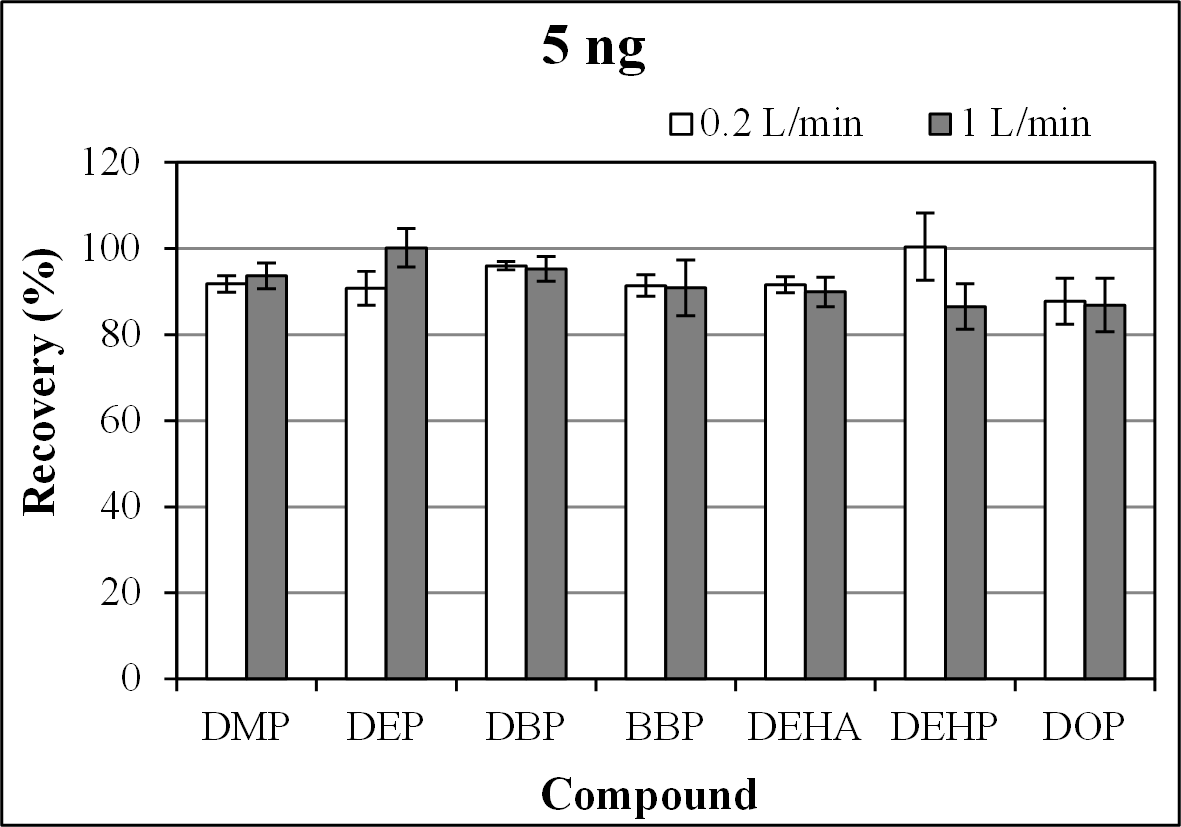

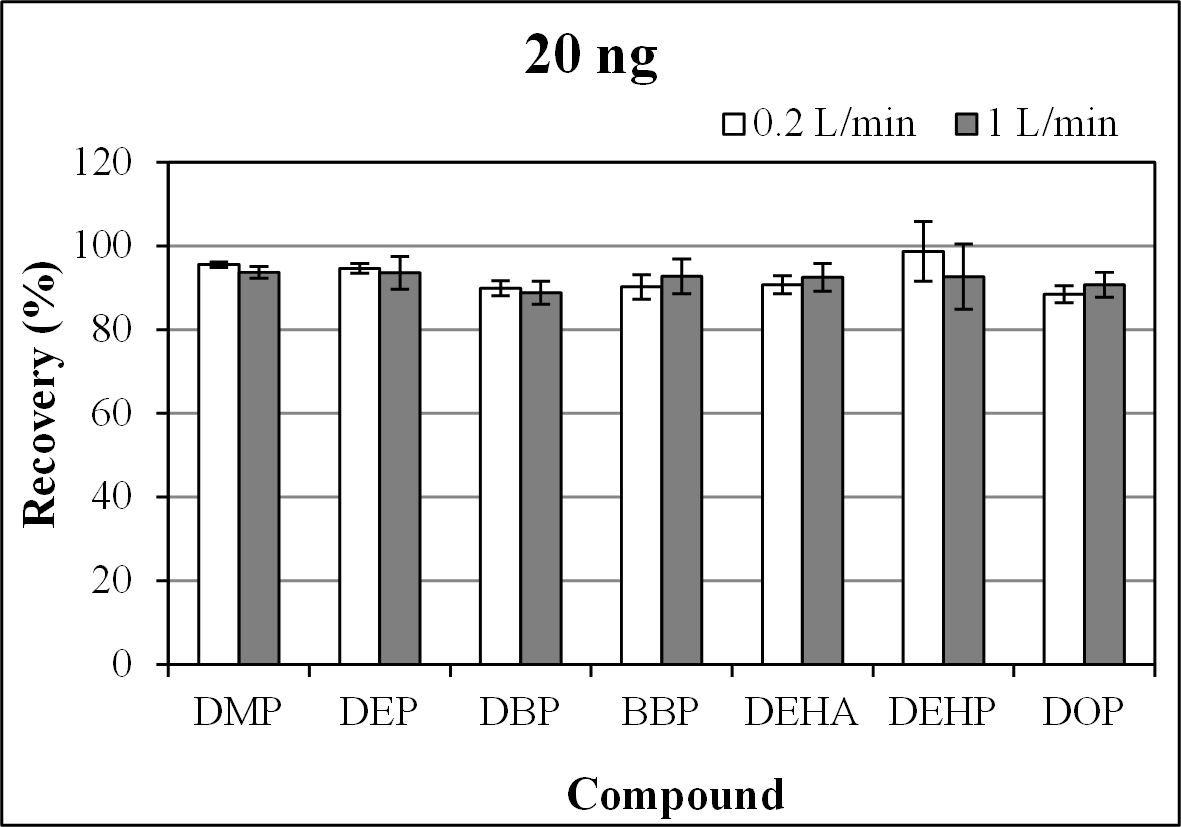

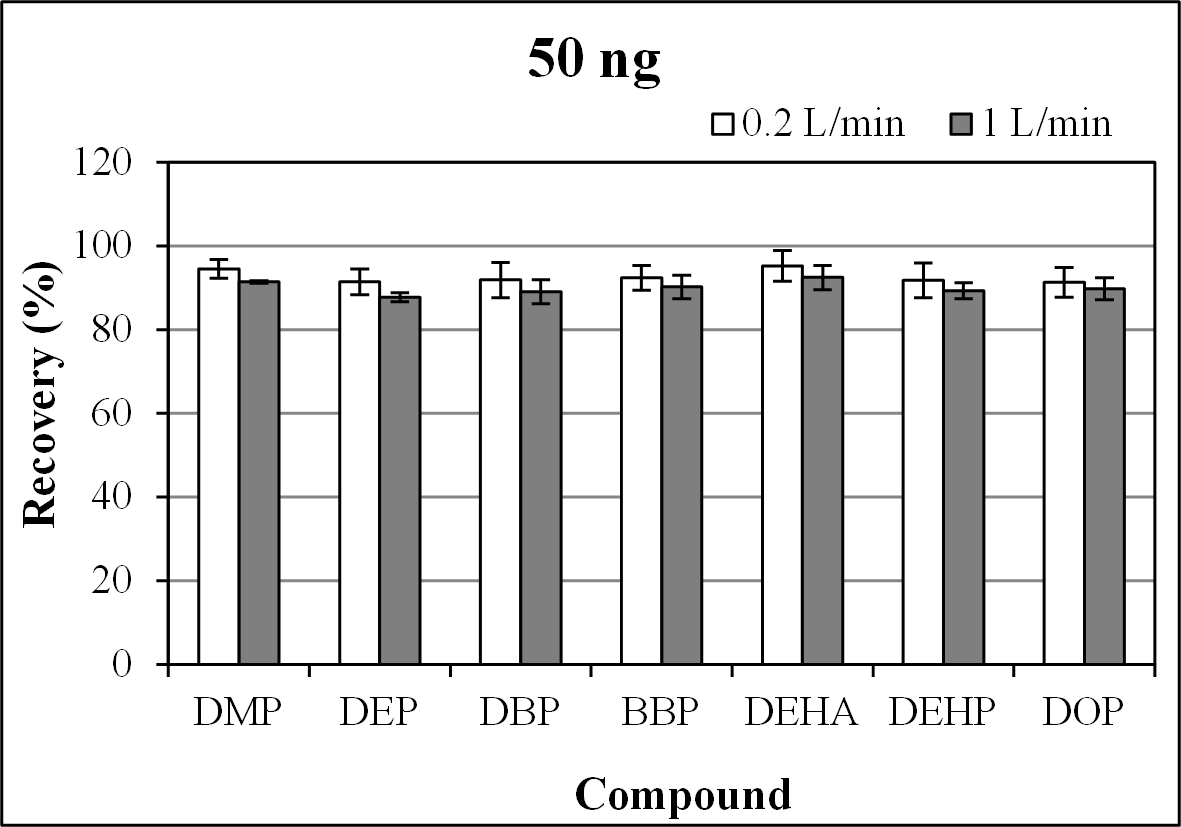


(B) Glass wool (GW) tube


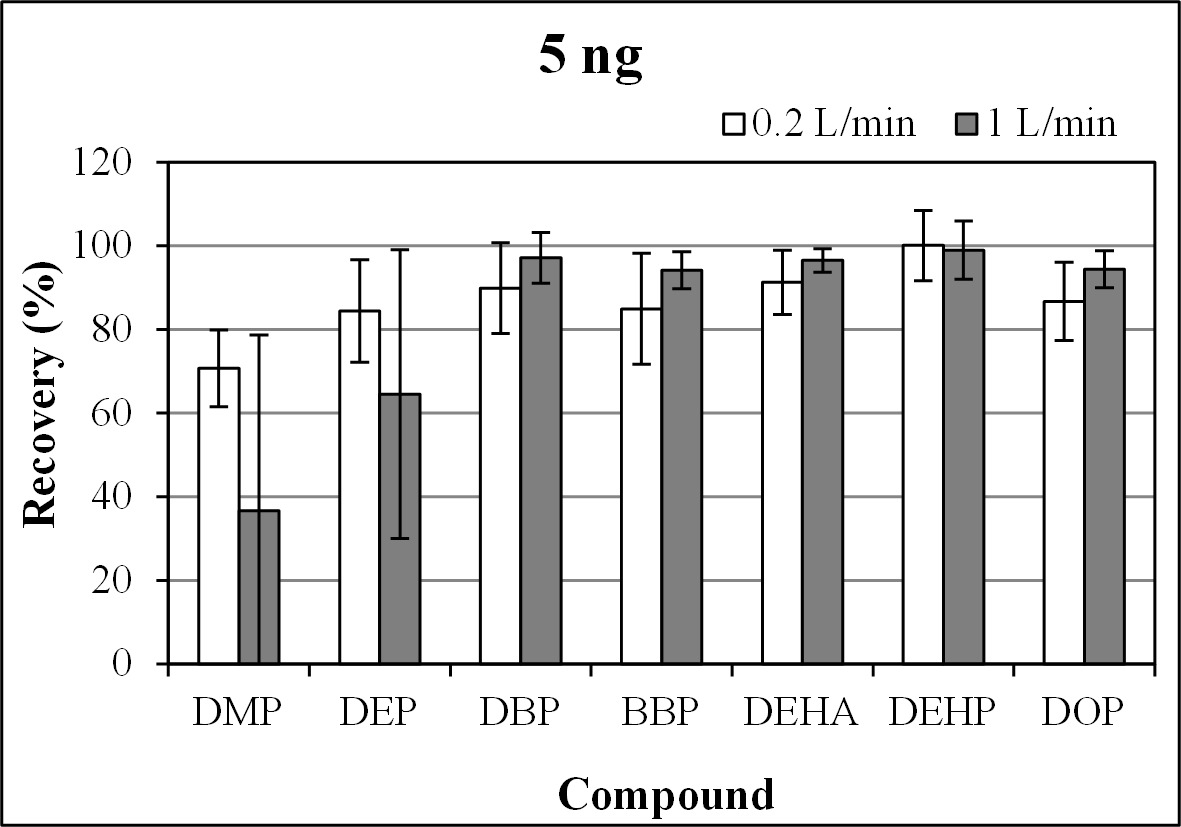

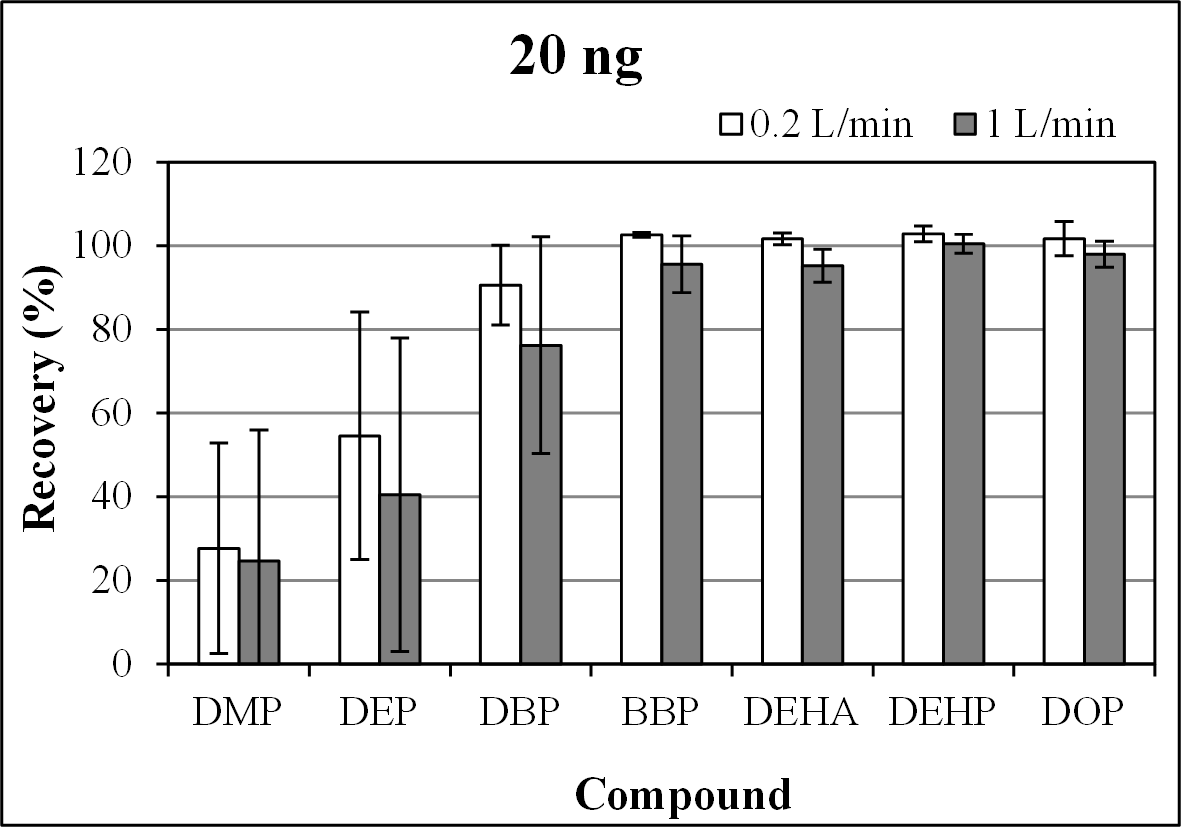

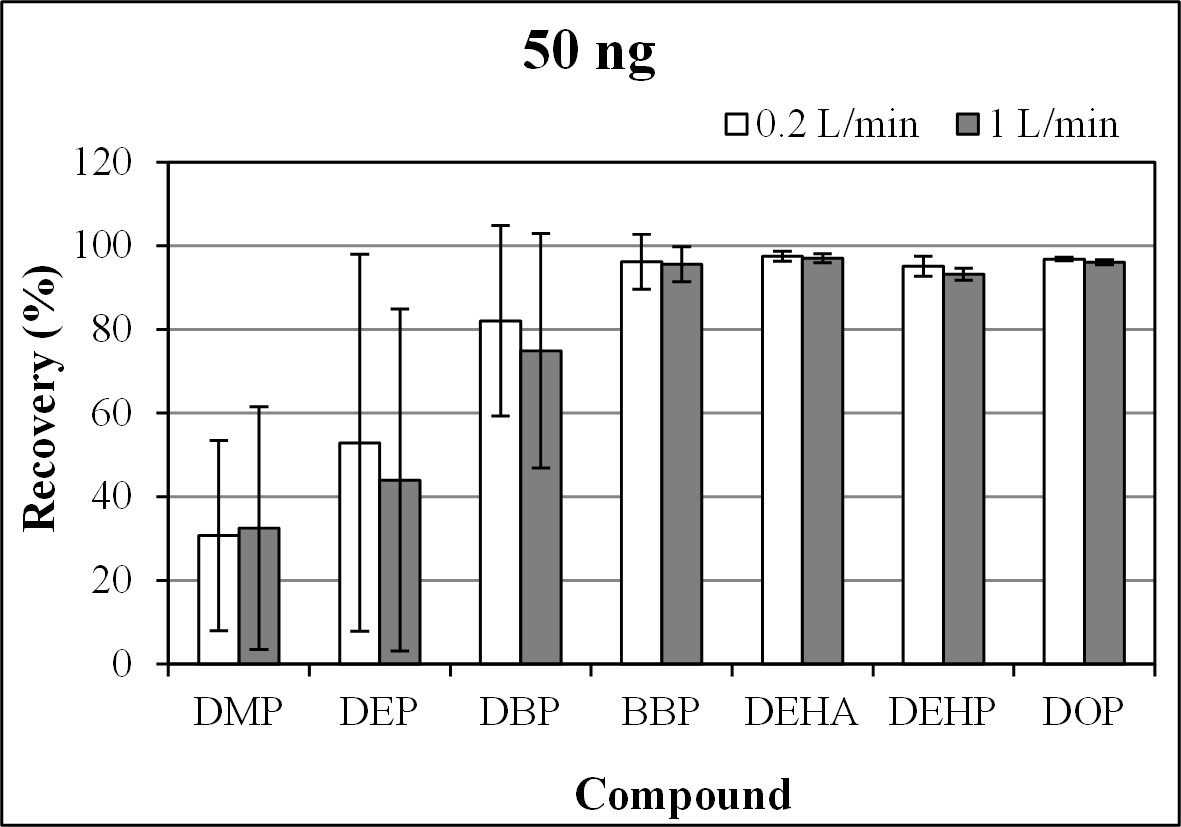


(C) Quartz wool plus Tenax TA (QWTN) tube


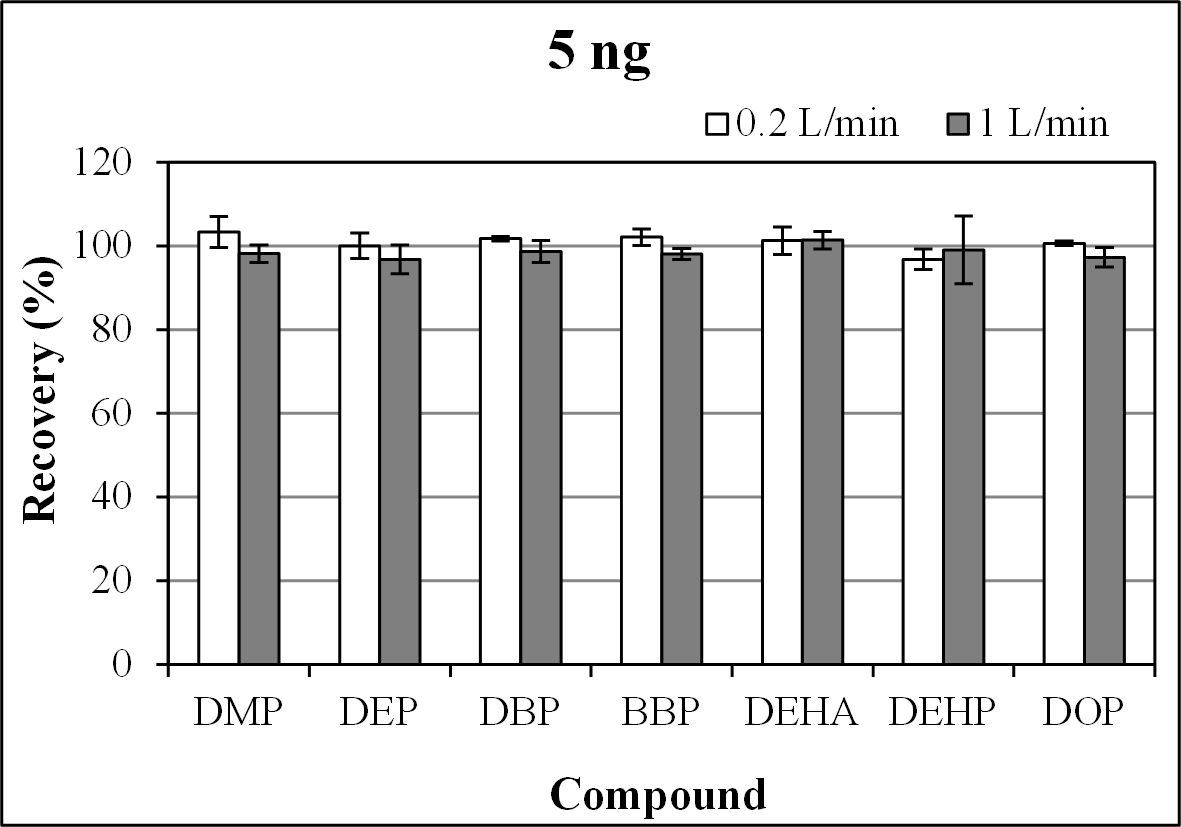

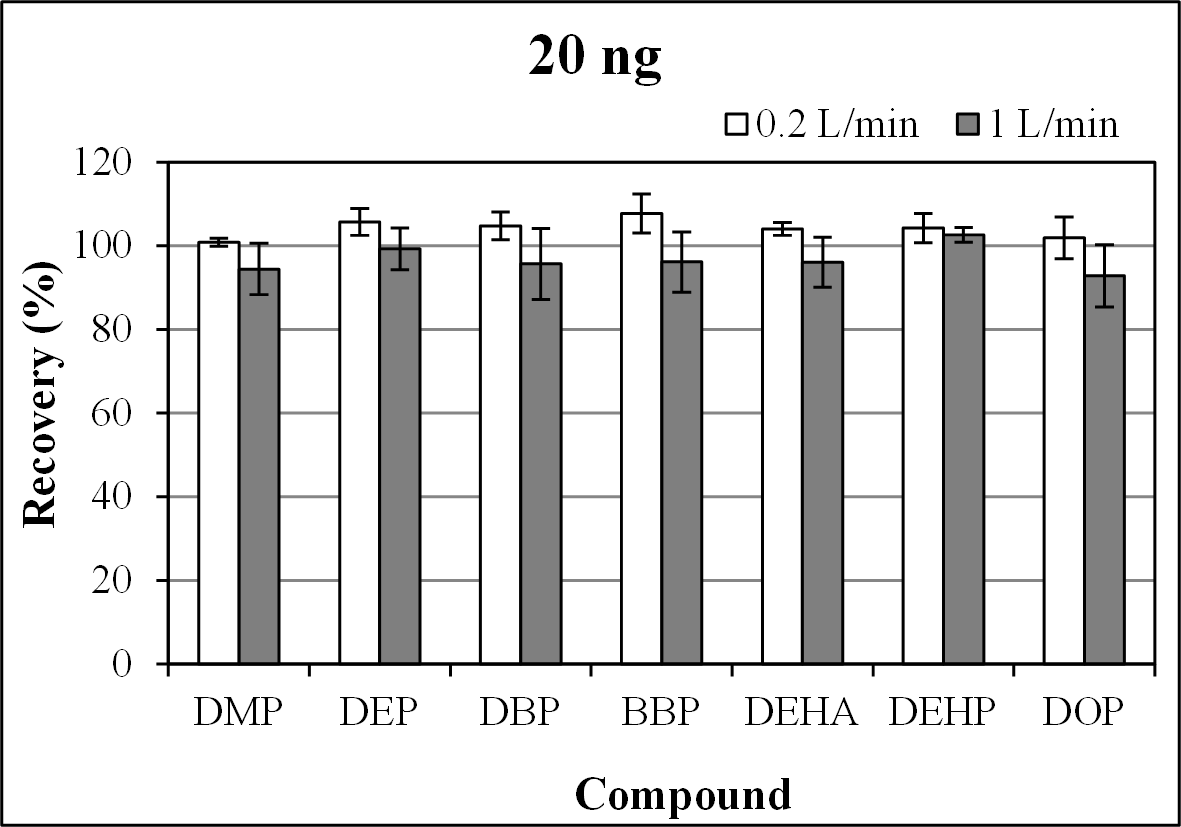

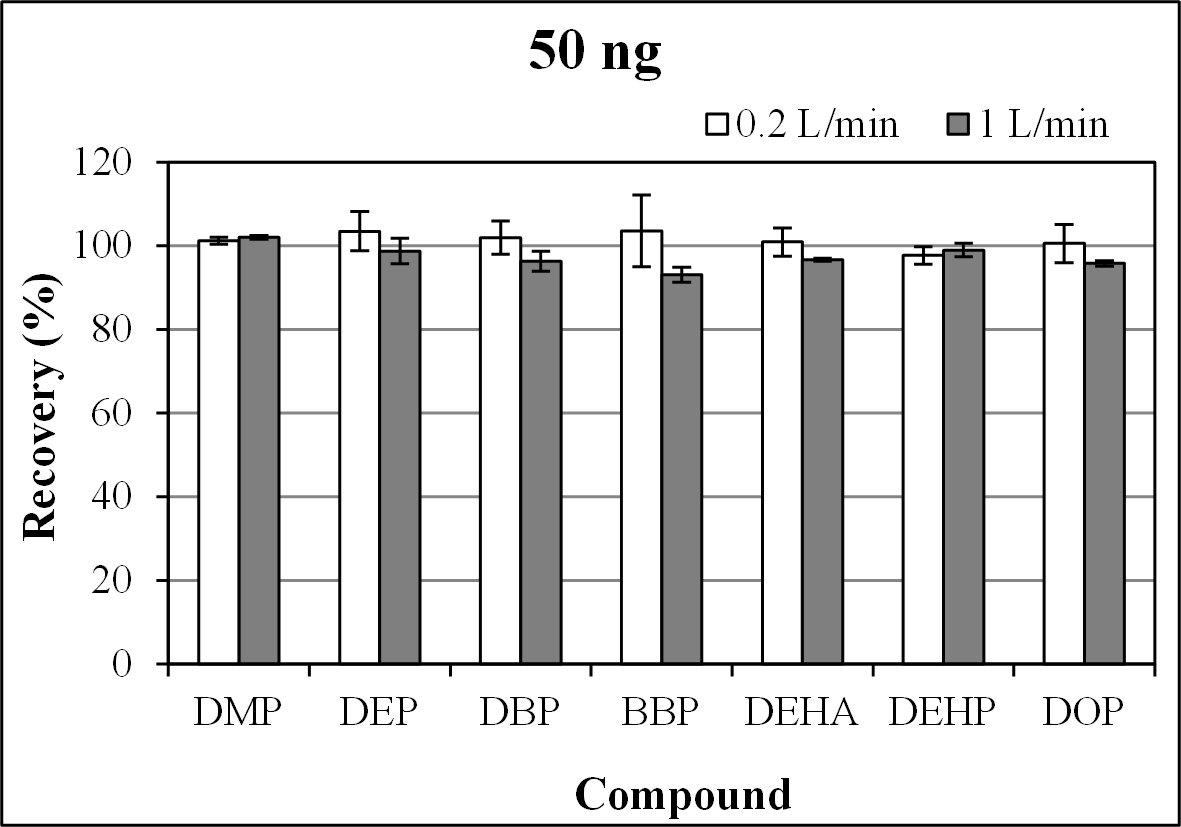


Figure S3. Comparison of average recovery (%) based on purge flow rate (0.2 and 1 L min^-1^).


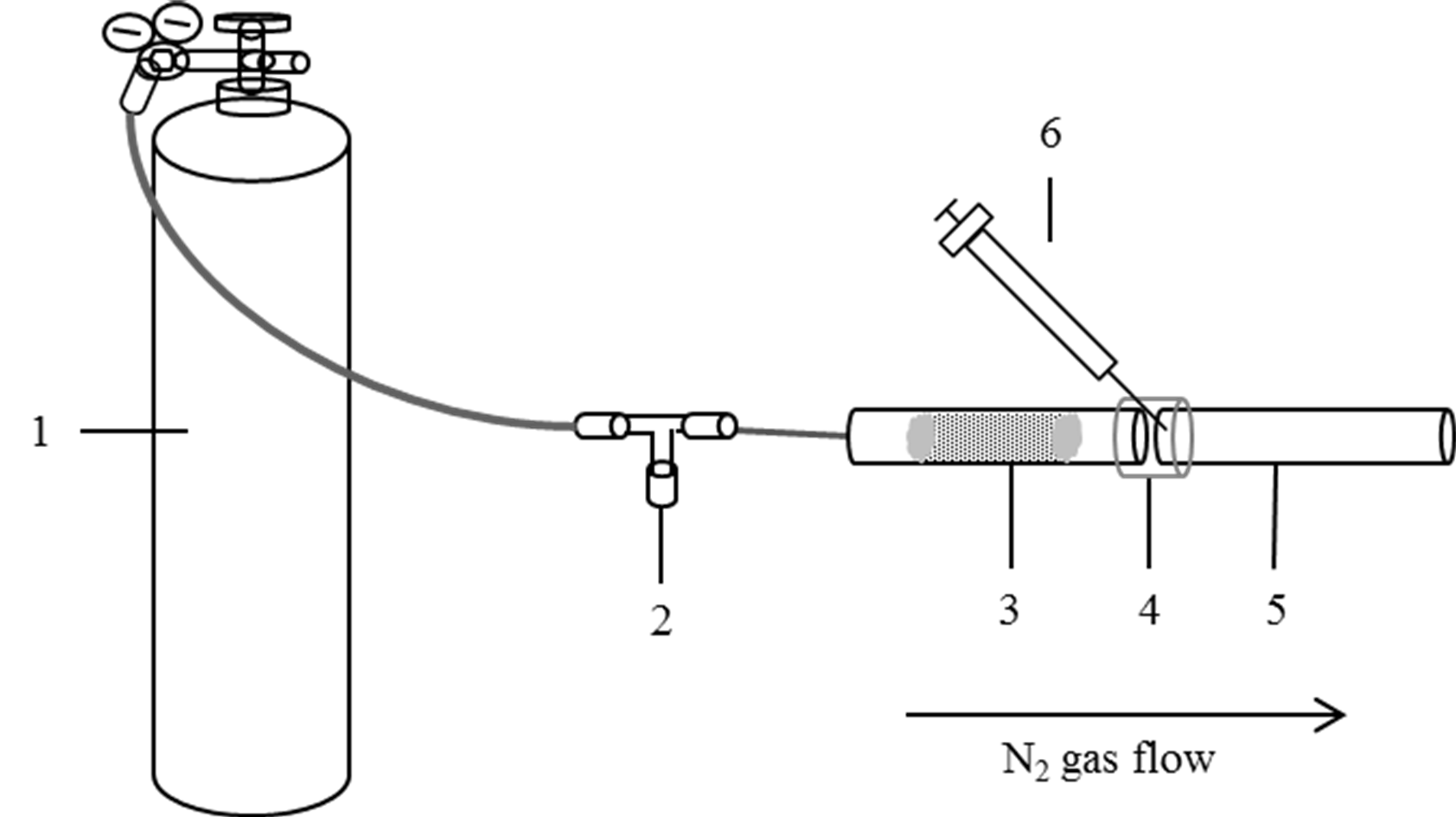


Figure S4. A schematic diagram for the collection of the vaporized liquid standard of phthalates by employing an N_2_ purge method: [1] Pure (99.999%) N_2_ cylinder; [2] Fine valve (flow rates of N_2_ gas were controlled at 0.2 or 1 L min^-1^; [3] Pre-filter (Carbopack X tube); [4] Teflon tubing; [5] Sorbent tube (QW, GW, and QWTN tubes); and [6] Liquid type syringe.
